# Comprehensive Approach for Sequential MALDI-MSI Analysis of Lipids, *N*-Glycans, and Peptides in Fresh-Frozen Rodent Brain Tissues

**DOI:** 10.1021/acs.analchem.4c05665

**Published:** 2025-01-09

**Authors:** Yea-Rin Lee, Ibrahim Kaya, Elin Wik, Sooraj Baijnath, Henrik Lodén, Anna Nilsson, Xiaoqun Zhang, Dag Sehlin, Stina Syvänen, Per Svenningsson, Per E. Andrén

**Affiliations:** † Department of Pharmaceutical Biosciences, Spatial Mass Spectrometry, Science for Life Laboratory, Uppsala University, SE-75124 Uppsala ,Sweden; ‡ Department of Public Health and Caring Sciences, Uppsala University, SE-75237 Uppsala ,Sweden; § Integrated Molecular Physiology Research Initiative, School of Physiology, Faculty of Health Sciences, University of the Witwatersrand, Johannesburg 2017, South Africa; ∥ Department of Clinical Neuroscience, Karolinska Institute, SE-17177 Stockholm ,Sweden

## Abstract

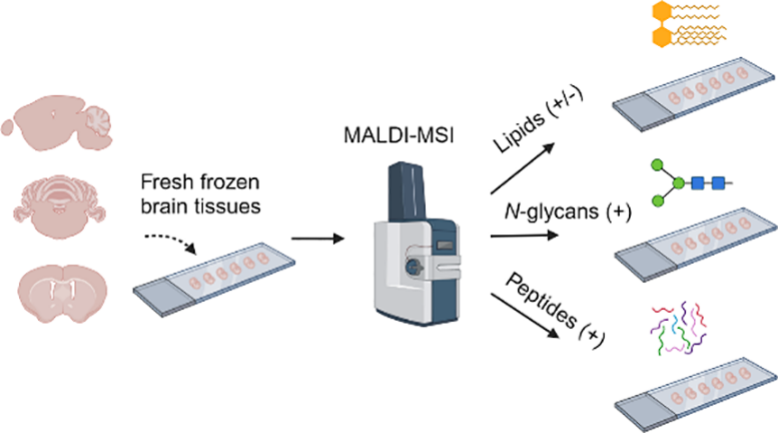

Multiomics analysis of single tissue sections using matrix-assisted laser desorption/ionization mass spectrometry imaging (MALDI-MSI) provides comprehensive molecular insights. However, optimizing tissue sample preparation for MALDI-MSI to achieve high sensitivity and reproducibility for various biomolecules, such as lipids, *N*-glycans, and tryptic peptides, presents a significant challenge. This study introduces a robust and reproducible protocol for the comprehensive sequential analysis of the latter molecules using MALDI-MSI in fresh-frozen rodent brain tissue samples. The optimization process involved testing multiple organic solvents, which identified serial washing in ice-cold methanol, followed by chloroform as optimal for *N*-glycan analysis. Integrating this optimized protocol into MALDI-MSI workflows enabled comprehensive sequential analysis of lipids (in dual polarity mode), *N*-glycans, and tryptic peptides within the same tissue sections, enhancing both the efficiency and reliability. Validation across diverse rodent brain tissue samples confirmed the protocol’s robustness and versatility. The optimized methodology was subsequently applied to a transgenic Alzheimer’s disease (AD) mouse model (tgArcSwe) as a proof of concept. In the AD model, significant molecular alterations were observed in various sphingolipid and glycerophospholipid species, as well as in biantennary and GlcNAc-bisecting *N*-glycans, particularly in the cerebral cortex. These region-specific alterations are potentially associated with amyloid-beta (Aβ) plaque accumulation, which may contribute to cognitive and memory impairments. The proposed standardized methodology represents a significant advancement in neurobiological research, providing valuable insights into disease mechanisms and laying the foundation for potential preclinical applications. It could aid the development of diagnostic biomarkers and targeted therapies for AD and other neurodegenerative diseases, such as Parkinson’s disease.

## Introduction

Matrix-assisted laser desorption/ionization mass spectrometry imaging (MALDI-MSI) has become a valuable tool for spatially resolved molecular analysis in pathological tissues. This versatile imaging technique allows the detection of hundreds of untargeted molecules and their spatial distribution within a single experiment, providing deep insights into the molecular heterogeneity of complex biological tissue samples. MALDI-MSI has been particularly influential in studying various diseases, including neurodegenerative disorders such as Alzheimer’s disease (AD) and Parkinson’s disease.^1−4^

The preparation of tissue sections for MALDI-MSI is critical to ensuring accurate and reproducible molecular analysis. Different tissue sample preparation strategies can be applied to investigate various molecules, as each provides unique biological information within tissue sections. Thus, combining this information as a multiomics approach enhances the understanding of complex molecular mechanisms of diseases at the tissue level. Fresh-frozen (FF) tissue samples are often considered optimal for MALDI-MSI as they maintain biological stability and are ideal for metabolomics and lipidomics analysis.^5^ However, there is also a high risk of molecule degradation and delocalization during sample preparation, particularly when enzymatic digestion is required for glycomics and peptidomics.

Formalin-fixed paraffin-embedded (FFPE) tissue samples are a viable alternative as they better preserve the tissue morphology and offer easier handling. However, FFPE tissue samples typically undergo extensive mechanical and chemical treatments during processing, which can result in a loss of crucial endogenous molecules. Moreover, the duration of formalin fixation — ranging from hours to days depending on tissue type and size — can introduce variability that may complicate reliable molecular imaging. Despite this, FFPE tissues have recently been used more frequently for glycomics^6^ and peptidomics^7^ MALDI-MSI analysis, as the processes of deparaffinization, antigen retrieval and enzymatic digestion are well-optimized and relatively less challenging than those needed to produce FF tissues.^8^

There is currently growing interest in performing multiomics analyses on a single tissue section.^9^ Previous studies have demonstrated the potential of spatial multiomics analyses using single FF tissue sections, while a similar study applied to FFPE tissue sections underscores the value of this approach.^10−12^ However, little attention has been paid to developing a comprehensive workflow that integrates multiomics approaches within a single tissue section, particularly for FF tissues, despite their suitability for analyzing diverse molecules.

In this study, we aimed to develop and validate a robust tissue washing protocol, which was then integrated into a comprehensive sequential MALDI-MSI analysis of lipids (in dual polarity mode), *N*-glycans and tryptic peptides within a single tissue section. The protocol was systematically optimized using various FF rodent control brain tissue sections and subsequently applied to investigate molecular alterations associated with amyloid pathology in a transgenic AD mouse model (tgArcSwe) as a proof of concept. The AD model was chosen as it is well-known for exhibiting distinct region-specific molecular alterations in brain tissue.

AD is the leading cause of dementia, affecting millions of people worldwide with a substantial societal and economic burden.^13^ The pathological hallmarks of AD include accumulation of amyloid-beta (Aβ) plaques and neurofibrillary tangles composed of phosphorylated tau proteins, which lead to synaptic dysfunction and neuronal loss.^13^ Consequently, disease-modulating strategies have historically focused on targeting these peptides. Conversely, the involvement of other molecules, such as lipids and *N*-glycans, has received less attention. However, these molecules have recently gained recognition as significant contributors to AD pathogenesis. Our optimized methodology for sequential multiomics analyses could provide deeper insights into AD and other neurodegenerative diseases, such as Parkinson’s disease. This approach may facilitate the discovery of novel biomarkers and therapeutic strategies.

## Experimental Section

### Chemicals and Reagents

All chemicals used in sample preparation were purchased from Sigma-Aldrich Sweden AB (Stockholm, Sweden) unless otherwise specified. High-purity chemicals suitable for mass spectrometry were selected. PNGase F was purchased from New England BioLabs (Ipswich, MA). Trypsin Gold was purchased from Promega Corporation (Madison, WI).

### Animal Experiments

Methodological development was conducted using a 12-week-old C57Bl/6 male mouse (control; CTRL, *n* = 1) and a 12-week-old Sprague–Dawley male rat (CTRL, *n* = 1). For proof-of-concept analysis, 16 to18-month-old C57BL/6 female mice (CTRL, *n* = 5) and age-matched transgenic mice with both the Arctic and Swedish amyloid precursor protein (APP) mutations (tgArcSwe; Alzheimer’s disease model; AD, *n* = 5) were used.^14^ All animal procedures were conducted in agreement with the European Communities Council Directive of November 24, 1986 (86/609/EEC) on the ethical use of animals and were approved by the local ethical committee, in compliance with both national and local animal care and use guidelines (Dnr 5.8.18–20401/20 at Uppsala University, Uppsala, Sweden, and N105/16 and N351/08 at Karolinska Institute, Stockholm, Sweden).

Brain tissue samples were cryo-sectioned sagittally or coronally using a Leica CM3050 S cryostat (Wetzlar, Germany) at −23 °C with a thickness of 12 μm. Sections were thaw-mounted onto indium tin oxide (ITO) coated glass slides (Bruker Daltonics, Bremen, Germany) and stored at −80 °C until MALDI-MSI analysis. For methodological development, each ITO slide contained at least two consecutive technical replicate sections of CTRL brain tissue from either a mouse or rat. For proof-of-concept analysis, AD (*n* = 5) and CTRL (*n* = 5) mouse brain tissue sections were placed together on a single ITO slide.

### Design of MALDI-MSI Experiments

A step-by-step approach was employed for method development. The optimized tissue wash method was initially tested on sagittal control mouse brain sections for *N*-glycan analysis, excluding lipid and tryptic peptide analysis. After confirming successful spatial distribution without delocalization and the absence of high lipid signals in the spectra, the fully integrated protocol was tested on coronal control rat cerebellum sections, before being applied to preclinical tissue samples, such as those from the transgenic AD mouse model (tgArcSwe). To assess the applicability and reproducibility of the method, different rodent brain tissue samples from various levels were selected. Each brain sample, containing at least two consecutive technical replicates, was processed and measured in alternating order. These consecutive technical replicate tissue sections were also used in a validation method by comparing their overall spectra and number of *m*/*z* peaks. As a proof of concept, coronal brain tissue sections from CTRL (*n* = 5) and AD (*n* = 5) model mice were placed on the same ITO slide to demonstrate molecular changes and their spatial distribution patterns.

### MALDI-MSI Sample Preparation

Prior to matrix application for dual polarity lipid analysis, brain tissue sections on an ITO slide were thawed in a vacuum desiccator for 15 min. A matrix solution containing 7.5 mg/mL norharmane dissolved in 80% methanol was deposited using a HTX M3-Sprayer (HTX Technologies, LLC, Chapel Hill, NC) (see Table S1 for spray-specific settings). The slide was marked at the edges with water-based white-out and scanned at 3200 dpi using a flatbed scanner (Epson Perfection V500, Nagano, Japan) for MALDI instrument teaching purposes.

Following MALDI-MSI imaging of lipids, the norharmane matrix was removed from the slide and delipidation was performed with serial washes in ice-cold 100% methanol (2 × 1 min) and 100% chloroform (2 × 30 s). The slide was then briefly dried in a vacuum desiccator for 1 min, followed by antigen retrieval in a water-bath (10 mM citric acid, pH 6.0) at 95 °C for 45 min prior to enzyme application. PNGase F (30 μL) diluted with LC-grade water (720 μL) was deposited using the M3 sprayer (see Table S1 for spray-specific settings). Following spraying, the slide was placed in a container with potassium sulfate (10.67 g of potassium sulfate in 3.64 g of LC-grade water) and incubated overnight at 37 °C in a humid chamber. The slide was marked at the edges with water-based white-out and scanned again. Subsequently, a 7 mg/mL α-cyano-4-hydroxycinnamic acid (CHCA) matrix solution in 50% acetonitrile (ACN)/0.1% (v/v) trifluoroacetic acid (TFA) was sprayed onto the tissue sections using the M3 sprayer (see Table S1 for spray-specific settings).

Following MALDI-MSI imaging of *N*-glycans, the CHCA matrix was removed and the slide was rehydrated with serial washes in 70% ethanol (2 × 30 s) and LC-grade water (30 s). The slide was then placed in a vacuum desiccator until fully dry. Lyophilized trypsin was reconstituted in LC-grade water immediately prior to usage to give a final concentration of 0.1 μg/μL. Trypsin deposition was performed using the M3 sprayer (see Table S1 for spray-specific settings). Subsequently, the slide was incubated in a humidity chamber for 4 h at 37 °C. Finally, the CHCA matrix solution was deposited using the M3 sprayer, following the optimized parameters indicated in Table S1. H&E staining was conducted after MALDI-MSI analysis and scanned for coregistration purposes.

### MALDI-MSI Data Acquisition

The MALDI-MSI experiments were conducted using a timsTOF fleX dual-source MALDI mass spectrometer (Bruker Daltonics) with a lateral resolution of 30 μm. Each experiment employed different acquisition parameters, as detailed in Table S2. Instrument calibration was performed using red phosphorus clusters in the *m*/*z* range of 0 to 2000 Da for negative mode and 0 to 3000 Da for positive mode. For lipid analysis, internal calibration was conducted using specific ion signals: *m*/*z* 834.5291 (PS (40:6); [M – H]^−^) for negative polarity analysis and *m*/*z* 760.5851 (PC (34:1); [M + H]^+^) for positive polarity analysis. Prior to the MSI experiment, the slides underwent height correction and focus adjustment.

### Data Processing

All MALDI-MSI data were analyzed using SCiLS Lab software (v.2023b Pro, Bruker Daltonics), with initial preprocessing involving TopHat baseline subtraction and normalization to root-mean-square. Brain regions were precisely annotated using optically scanned images aligned to the Allen Brain Atlas (https://mouse.brain-map.org/). Since the method development analysis was conducted only using CTRL tissues (*n* = 1 for both a mouse and a rat), no statistical analysis was performed. Instead, selected *m*/*z* peaks that were previously validated and published were assessed for each molecule type (i.e., lipids, *N*-glycans and tryptic peptides) to ensure the protocol’s applicability, versatility and image quality. Additionally, spatial segmentation using the Bisecting k-Means clustering algorithm in SCiLS was conducted to validate the method’s consistency across consecutive technical replicate brain tissue sections and to determine region-specific differences within the tissue samples.

In preclinical samples comprising both CTRL and AD groups, putative *m*/*z* peak lists with normalized spectral intensities for each molecule type were generated using sliding window functions in SCiLS. These lists, corresponding to each annotated region, i.e., cerebral cortex (CTX), corpus callosum (CC), caudoputamen (CP), nucleus accumbens (NAc) and lateral septal complex (LS), were then exported into Excel files for subsequent statistical analyses. Statistical analyses were performed using GraphPad Prism (v.10, Boston, MA). As part of the untargeted analysis, combined normalized putative *m*/*z* intensity lists from all molecular data sets were used to generate volcano plots for each annotated brain region. These plots were based on log_2_ fold changes and –log_10_*t*-test *p*-values, with thresholds set at *p* < 0.05 (equivalent to 1.3010 on the −log_10_*p*-value scale) and fold changes >2 (corresponding to ±1 on the log_2_ fold change scale). This approach allowed the extraction of putative *m*/*z* features that were significantly different between the groups. Selected putative features were then manually evaluated for their spatial distribution patterns and matched against databases for identification (detailed explanation follows). Nonrelevant features were excluded from the final list. From this refined list, the top 20 *m*/*z* features showing the most significant differences between the CTRL and AD groups were selected for further statistical analysis. Log-transformed intensity values were used for these analyses. Data normality was initially assessed using the Shapiro-Wilk test. For normally distributed data, unpaired *t*-tests with Welch’s correction were used, whereas Mann–Whitney tests were conducted for non-normally distributed data. Statistical significance was set at *p* < 0.05. For heatmap generation, the normalized intensities of the top 20 *m*/*z* features were converted to *z*-scores. The row *z*-score value of each feature was calculated as the difference between the mean intensity and individual intensity, divided by the standard deviation across all samples.

Lipids were initially annotated in MALDI-MSI data by matching accurate mass measurements (within a ±3 ppm mass error tolerance) to the Lipid Maps database (https://www.lipidmaps.org/).^15^ To confirm these identifications, the distributions of sodium and potassium adducts of the same lipid species were compared with their corresponding [M + H]^+^ ions and cross-referenced with MALDI-FT-ICR-MSI data from comparable brain regions in a previous study.^4^ For *N*-glycans, selected putative *N*-glycans were searched using the UniCarb database (https://unicarb-db.expasy.org/) *via* GlycoMod (http://web.expasy.org/glycomod/) for their monosaccharide composition and structure.^16^ Only *N*-glycans detected as [M + Na]^+^ and found on the GlyConnect database were included in the statistical analyses. Further confirmation of lipid and *N*-glycan identities was achieved by comparison with published data through mass matching and/or on-tissue MS/MS collision-induced dissociation (CID) fragmentation. On-tissue MS/MS focused on specific brain regions with abundant target ions and was performed using either a MALDI-FT-ICR (Solarix XR 7T-2Ω, Bruker Daltonics) or timsTOF flex (Bruker Daltonics).^3,4,17−20^ On-tissue MS/MS was conducted only when the corresponding peak exhibited sufficient intensity for isolation and fragmentation. The collision energy was optimized for each analyte.

Due to challenges in identifying tryptic peptides *via* on-tissue MS/MS fragmentation, confirmation was achieved through a tissue in-solution digest method optimized in-house for liquid chromatography-tandem mass spectrometry (LC-MS/MS). Details of the sample preparation, data acquisition, and analysis are provided in the Supporting Information. Singly charged *m*/*z* values from MALDI-MSI peptide data sets were manually cross-referenced with doubly charged LC-MS/MS peptides using a tolerance of ±7 ppm. Further validation of tryptic peptide identities was supported by comparison with published data.^21,22^

### Aβ Immunostaining

To confirm the presence of Aβ pathology in tgArcSwe mice, consecutive sections adjacent to the MALDI-imaged brain sections were used for Aβ40 immunostaining. CTRL brain sections were also included for comparison. Tissue sections were fixed with 4% paraformaldehyde for 20 min, followed by antigen retrieval in preheated citrate buffer (25 mM, pH 6.0) for 40 min at room temperature and a 5 min incubation in 70% formic acid (FA). Permeabilization was performed with 0.4% Triton X-100 in PBS, followed by blocking with 5% normal goat serum (Bionordika, Solna, Sweden) and a brief incubation in 0.1% PBS Tween-20. The sections were incubated overnight at +4 °C with the primary antibody anti-Aβ40 (1:500; Invitrogen, Carlsbad, CA) and the next day with Alexa Fluor 488 goat-antirabbit secondary antibody (1:500; A11029, Sigma-Aldrich, Saint Louis, MO) for 1 h at room temperature. Slides were mounted with Vectashield Antifade with DAPI (Vector Laboratories, Newark, CA) and imaged using a Zeiss Observer Z.1 microscope with ZEN 2.6 software (Carl Zeiss Microimaging GmbH, Jena, Germany).

## Results and Discussion

### Optimizing Tissue Washing Protocols for Sequential Multiomics MALDI-MSI Analysis

To enhance the spatial resolution of ion images and improve the detection of low concentration target molecules, we initially focused on optimizing the tissue washing protocol for *N*-glycan analysis. Given that approximately 60% of the brain’s dry weight consists of lipids, particularly sphingolipids, glycerophospholipids and cholesterol, it was crucial to remove these lipids without delocalizing other molecules before integrating the protocol into multiomics MALDI-MSI workflows.^23−25^ Initially, we explored various organic solvents for immersing FF brain tissue sections at different temperatures and durations. Based on existing literature and our previous experience, we selected common wash solvents such as ethanol, methanol, chloroform and Carnoy’s solution for testing^26,27^ (see Table S3 for further details). The combination of these solvents was carefully optimized to ensure that the washing process preserved the integrity of the tissue sections and the spatial distribution of the molecules, which served as the primary criterion for protocol evaluation.

**Figure 1:**
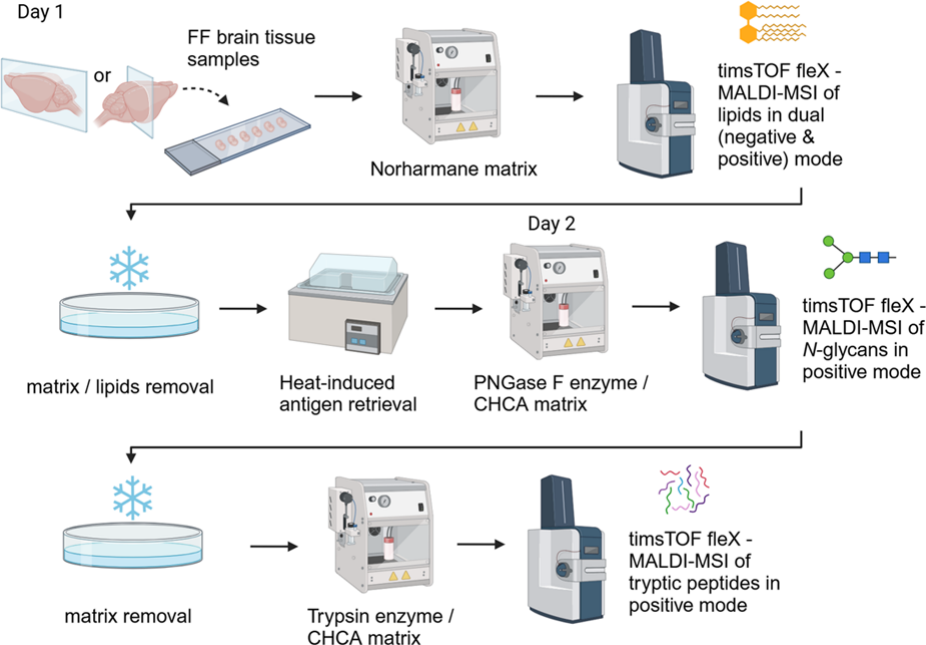
Overview of the multiomics MALDI-MSI workflow. Optimized protocols for sequential analysis of lipids (in dual polarity mode), *N*-glycans, and tryptic peptides on FF brain tissue sections mounted on an ITO slide. The entire process, from sample preparation to data acquisition, can be completed within 2 days.

For *N*-glycan analysis, we found that using a combination of ice-cold 100% methanol (2 × 1 min) followed by 100% chloroform (2 × 30 s) in a glass Petri dish yielded optimal results. Both methanol and chloroform are widely recognized as “gold standards” for lipid removal or extraction.^28,29^ When applied individually under ice-cold conditions, these solvents preserved tissue integrity while effectively removing lipids and preventing *N*-glycan delocalization (Figure S1). This approach enables the duration of tissue washing to be adjusted based on the tissue’s thickness and size. The optimized tissue washing protocol was also tested for tryptic peptide analysis using FF mouse cerebellum tissue sections and was found to achieve the same image quality (data not shown). These results demonstrated the protocol’s applicability and versatility. Once the tissue washing protocol was optimized, we integrated it into MALDI-MSI workflows to further develop sequential multiomics analyses. Utilizing state-of-the-art MALDI instrumentation and our refined protocol, we successfully performed sequential MALDI-MSI analysis of lipids (in dual polarity mode), *N*-glycans and tryptic peptides within a two-day time frame (Figure 1).

### Enhancing Applicability and Reproducibility in Optimized MALDI-MSI Workflows

To ensure the applicability and reproducibility of our optimized workflows, we implemented a systematic approach during method development. The initial focus was on optimizing the tissue washing protocol for *N*-glycan analysis to effectively remove lipids and minimize ion suppression without compromising molecular integrity. Once refined, the protocol was extended to enable the simultaneous analysis of lipids (in dual polarity mode), *N*-glycans and tryptic peptides. The procedure was tested on control rat cerebellum sections and then applied to preclinical tissue samples from the transgenic AD mouse model (tgArcSwe) as a proof of concept.

**Figure 2:**
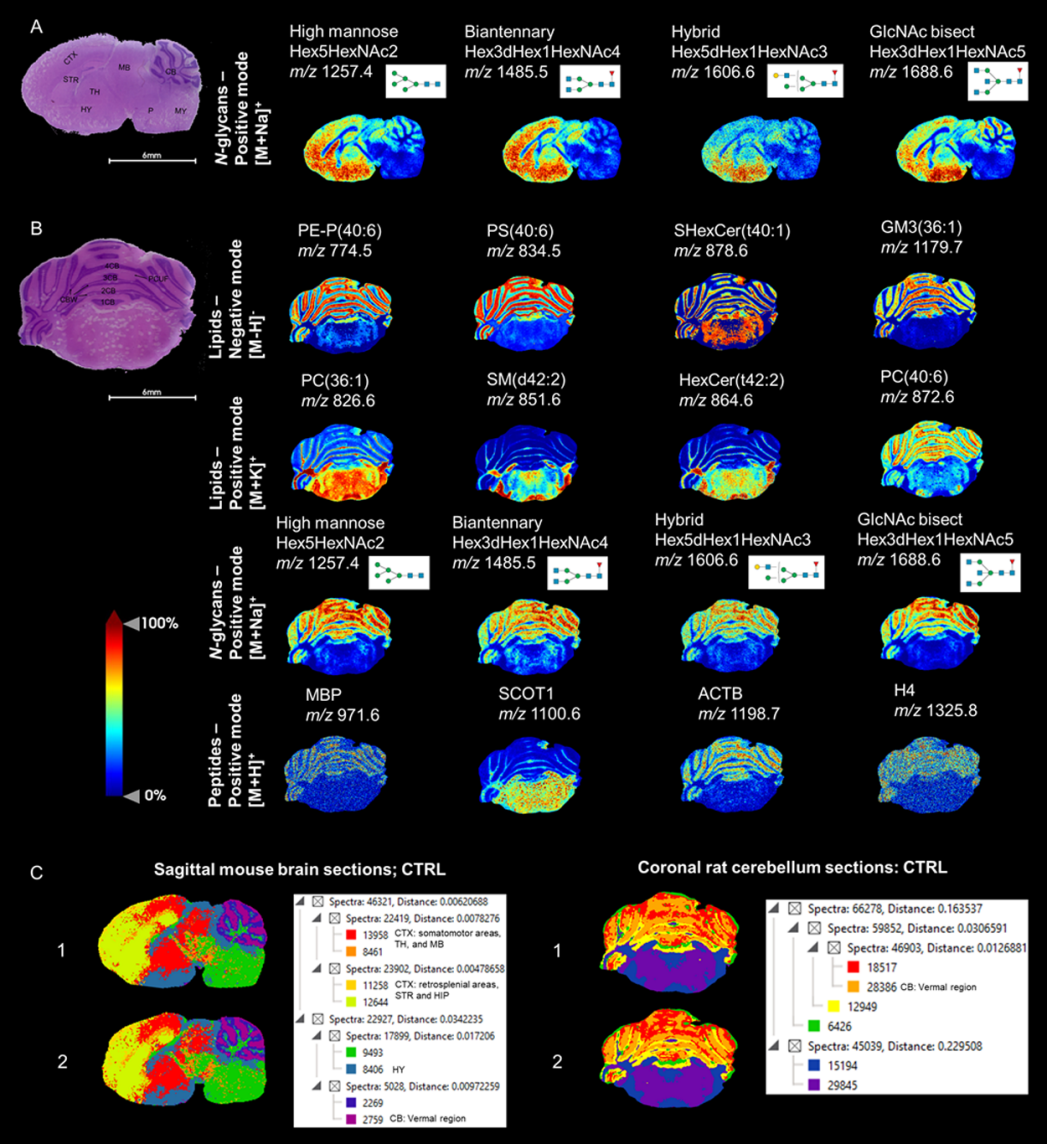
Validation of the optimized MALDI-MSI workflow across rodent brain tissue samples. (A) Sagittal control mouse brain sections: the optimized washing protocol effectively removed endogenous lipids while preserving *N*-glycan integrity, ensuring minimal sample delocalization for MALDI-MSI analysis. (B) Coronal control rat cerebellum sections: successful application of the optimized protocol on rat cerebellum sections demonstrated its versatility across different brain regions and species for comprehensive molecular analysis. (C) Bisecting k-means segmentation map: a representative image showing the segmentation map generated from the *N*-glycan MALDI-MSI data set on consecutive technical replicate brain tissue sections, with distinct clustering groups corresponding to specific brain regions. Additional H&E-stained images with annotated regions are provided in Figure S3. Abbreviations: CTX, cerebral cortex; STR, striatum; HIP, hippocampal region; TH, thalamus; HY, hypothalamus; MB, midbrain; CB, cerebellum; MY, medulla; P, pons; CBW, cerebellar white matter; 1CB, lobule 1 of cerebellar vermis; 2CB, lobule 2 of the cerebellar vermis; 3CB, lobule 3 of the cerebellar vermis; 4CB, lobule 4 of the cerebellar vermis; PCUF, preculminate fissure; MBP, myelin basic protein; SCOT1, succinyl-CoA:3-ketoacid coenzyme A transferase 1; ACTB, actin, cytoplasmic 1; H4, histone H4. Symbols for *N*-glycan monosaccharides: green circle—mannose, yellow circle—galactose, blue square—*N*-acetylglucosamine (GlcNAc), yellow square—*N*-acetylgalactosamine (GalNAc), and red triangle—fucose.

To validate its robustness, we utilized the protocol on various rodent brain tissue samples, confirming its effectiveness across different species. Representative molecules were used to illustrate region-specific spatial distributions, demonstrating the absence of delocalization (Figure 2). The comprehensive workflow, integrating optimized tissue washing with multiomics analysis — enabled the reliable biomolecular mapping of FF brain tissue sections (Figures 2, S1, S2, and S3).

Additionally, spatial segmentation maps generated using the Bisecting k-Means algorithm, which clusters MSI pixels based on mass spectral similarities, validated our methodology. These maps consistently grouped spectra into distinct brain regions across consecutive technical replicate tissue sections, emphasizing the robust reproducibility of our methodology (Figure 2).

### Comparative Molecular Profiling between AD and CTRL Brains

**Figure 3:**
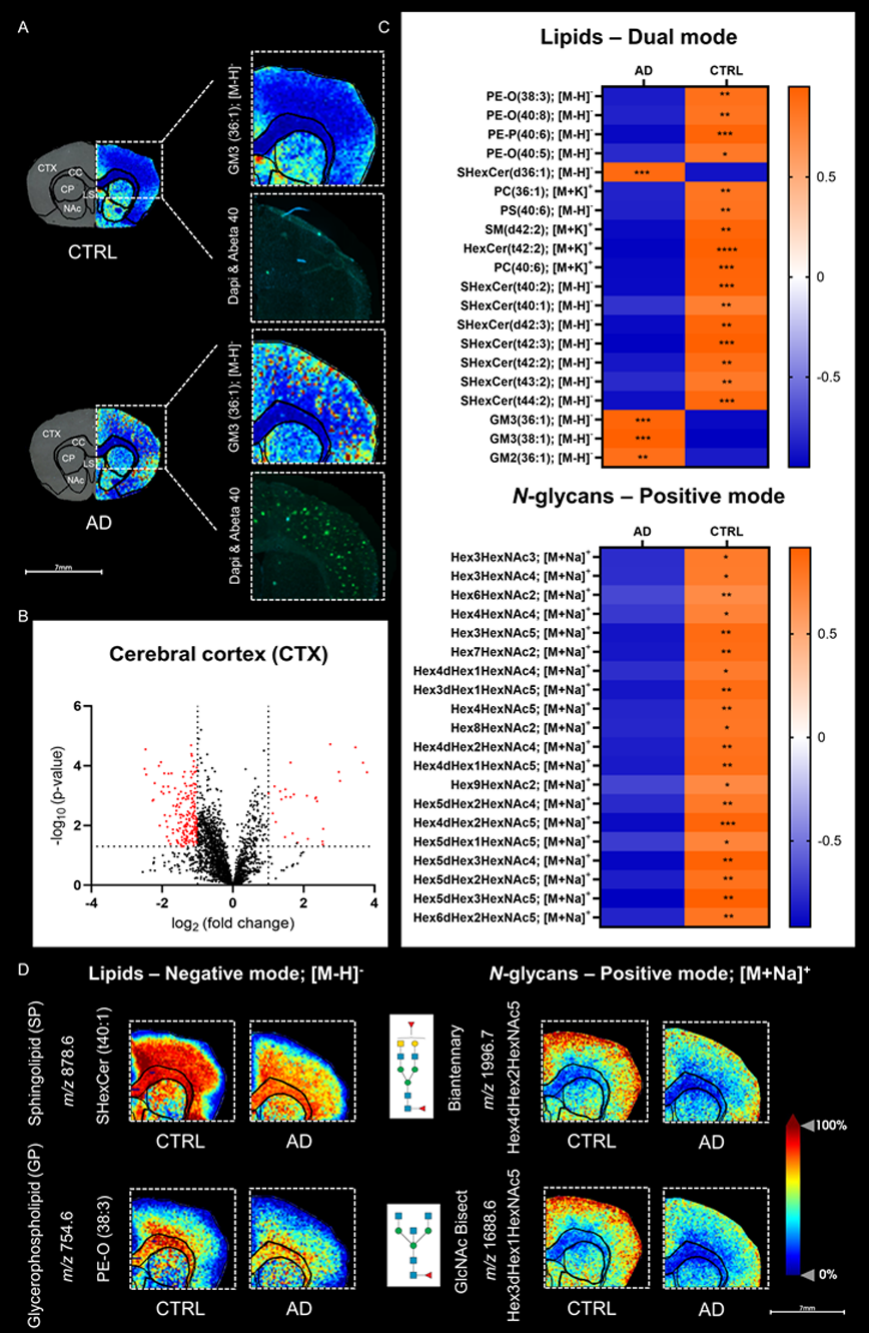
MALDI-MSI analysis revealing significant molecular alterations in the transgenic AD mouse model (tgArcSwe) compared to the CTRL group. (A) Representative ion image of GM3 (36:1) demonstrating a distinct distribution around Aβ plaques in the AD mouse model, with no corresponding presence in the CTRL. This observation was supported by a consecutive Aβ-immunostained tissue image, which showed Aβ plaques in the cerebral cortex (CTX). (B) Volcano plot illustrating the abundance of individual *m*/*z* features (combining lipid, *N*-glycan, and tryptic peptide data sets) and their fold changes (log_2_ scale) between the AD model and CTRL group, along with corresponding *p*-values (−log_10_ scale). The plot highlights 477 putative *m*/*z* values from lipids and *N*-glycans that were significantly altered in the CTX (>1.3 fold change, *p* < 0.05), marked in red. Additional volcano plots are provided in Figure S4. (C) Heatmaps generated using *z*-scores for the top 20 most significant *m*/*z* features of lipids and *N*-glycans between the groups. Orange indicates high abundance, whereas blue indicates low abundance. Statistical significance: * *p* < 0.05, ** *p* < 0.01, *** *p* < 0.001, **** *p* < 0.0001 (see Figure S6 for further details). (D) Representative ion images for selected lipids (i.e., SHexCer from the SP species and PE from the GP species) and *N*-glycans (i.e., biantennary and GlcNAc-bisecting). The selected lipids and *N*-glycans were among the top 20 most significantly different between the groups. Additional representative ion images for the top 20 *m*/*z* features are provided in Figure S6. Abbreviations: PE, phosphatidylethanolamines; PC, phosphatidylcholines; PS, phosphatidylserines; SHexCer, sulfohexosylceramides (sulfatides); HexCer, hexosyl ceramides; SM, sphingomyelins; GM, gangliosides. Symbols for *N*-glycan monosaccharides: green circle—mannose, yellow circle—galactose, blue square—*N*-acetylglucosamine (GlcNAc), yellow square—*N*-acetylgalactosamine (GalNAc), and red triangle—fucose.

We implemented the optimized protocol to analyze samples from a transgenic AD mouse model (tgArcSwe), characterized by heterogeneous Aβ plaques, and compared them to the CTRL group as a proof of concept (Figure 3).^30^ To investigate specific molecular changes between the groups, we performed an untargeted analysis by generating volcano plots based on the combined normalized intensities of putative *m*/*z* peaks across all molecular data sets for each brain region, i.e., cerebral cortex (CTX), corpus callosum (CC), caudoputamen (CP), nucleus accumbens (NAc) and lateral septal complex (LS). Plots above the nonaxial horizontal lines represented significantly altered *m*/*z* features (>1.3 fold change, *p* < 0.05), aiding in the identification of key molecular differences between the groups in each region.

Subsequently, we found 477 putative *m*/*z* features from lipids and *N*-glycans that were significantly altered in the CTX of the AD mouse model compared to the CTRL group, a region associated with amyloid plaque accumulation in AD (Figure 3). These changes were predominantly observed in the CTX compared to other regions, such as the CC, CP, NAc and LS (Figure S4), indicating that the CTX is more vulnerable to AD-related alterations. Among these, the top 20 most significant *m*/*z* features were selected for further statistical analysis, following a thorough evaluation of their spatial distribution patterns and subsequent identification against database matching and on-tissue MS/MS fragmentation (Figure 3, Table S4, and Figure S5). No significant differences were observed for tryptic peptides.

In the lipid profile analysis, we selected and identified the top 20 distinct lipid species in the dual mode, comprising 13 sphingolipid (SP) and 7 glycerophospholipid (GP), which showed differential expression between the AD and CTRL brains (Figures 3, S6 and S7). Of these, the SP class exhibited the most significant differences, particularly within the sulfatides (SHexCer, *n* = 8) subclass (Table S4). The GP class, especially the phosphatidylethanolamines (PE, *n* = 4) subclass, revealed the second most significant differences between the AD model and CTRL group (Table S4).

In the sequential *N*-glycan analysis, we selected and identified the top 20 most significant *N*-glycans between the groups, including 8 biantennary, 7 GlcNAc-bisecting, 4 high mannose and 1 multiantennary (Figures 3, S6 and S7). Among these, biantennary *N*-glycans, notably those with fucose, showed the most significant differences between the groups (Table S4). GlcNAc-bisecting *N*-glycans with fucose were the second most significant (Table S4).

### Cerebral Cortex-Specific Molecular Alterations in AD Model Brains

**Figure 4:**
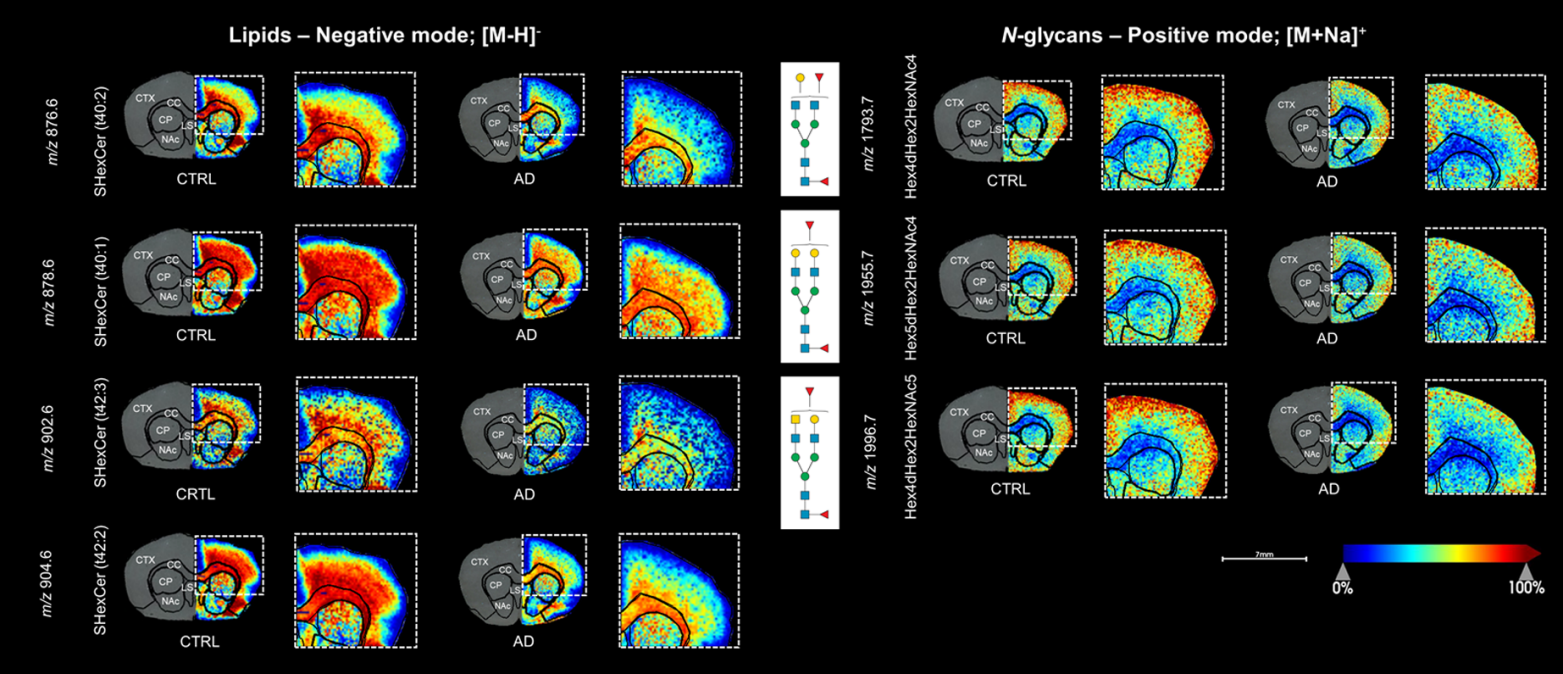
Disrupted SPs and biantennary *N*-glycans in the CTX of the transgenic AD mouse model. Specific molecular alterations were observed in SHexCer species and biantennary fucosylated *N*-glycans, predominantly localized in the CTX, indicative of pathological changes associated with AD. Additional individual ion images for the CTRL (*n* = 5) and AD (*n* = 5) groups are provided in Figure S7. Symbols for *N*-glycan monosaccharides: green circle—mannose, yellow circle—galactose, blue square—*N*-acetylglucosamine (GlcNAc), yellow square—*N*-acetylgalactosamine (GalNAc), and red triangle—fucose.

In the CTX of AD model brains, we observed specific alterations in lipid composition, particularly in long-chain hydroxylated SPs, such as SHexCer (t40:1), SHexCer (t40:2), SHexCer (t42:2) and SHexCer (t42:3) (Figures 4 and S8). These lipids showed a significant reduction in the AD model, especially in the inner layer of the CTX (Figures 4 and S8). Conversely, short-chain nonhydroxylated SPs, such as SHexCer (d36:1), and gangliosides (GM), including GM2 (36:1), GM3 (36:1) and GM3 (38:1), were more abundant in the CTX of the AD model compared to the CTRL, with a notable distribution around Aβ plaques (Figure S7). These findings suggest potential disruptions in lipid metabolism within the CTX region that are linked to amyloid plaque pathology and may contribute to neuronal and synaptic loss. This is consistent with previous studies reporting localized reductions in SPs and GPs around Aβ-positive plaques in the transgenic AD mouse model.^11,31−34^

SHexCer depletion causes a gradual deterioration of myelin with age, especially in aged transgenic models.^35,36^ The accelerated degradation of sulfatides appears to be associated with impaired apolipoprotein E-related Aβ clearance, contributing to fibril accumulation and AD progression.^37,38^ Interestingly, previous studies have shown that sulfatide levels are significantly depleted in AD, with no further decrease as the disease progresses.^39,40^ This implies that the sulfatide content is permanently altered early in AD and remains lower than in normal CTRLs. Additionally, the elevated GM3 levels observed in the brains of the AD model were supported by previous findings in the entorhinal cortex of post-mortem brain tissue from late-onset AD patients and in the transgenic AD mouse model.^41,42^ GMs, including GM3, are known to promote the amyloidogenic processing of APP, Aβ aggregation and the development of amyloid plaques.^43^

Fewer *N*-glycans were detected by MALDI-MSI in mouse brain tissue compared to lipids, likely due to the brain’s high lipid content. However, most of the *N*-glycans that differed between CTRL and transgenic AD brains had similar structures, primarily belonging to the biantennary and GlcNAc-bisecting *N*-glycans with fucose (Figure S7). This finding agrees with a previous study reporting that over 70% of *N*-glycans in the cortex were complex-type structures predominantly decorated with fucose, suggesting involvement of fucosyltransferases (FUT) in AD pathogenesis.^44^ The upregulation of FUT8 expression may be independent of the antioxidative response, and the resulting increase in fucosylation may provide a protective mechanism for cells under oxidative stress conditions.^45^

**Figure 5:**
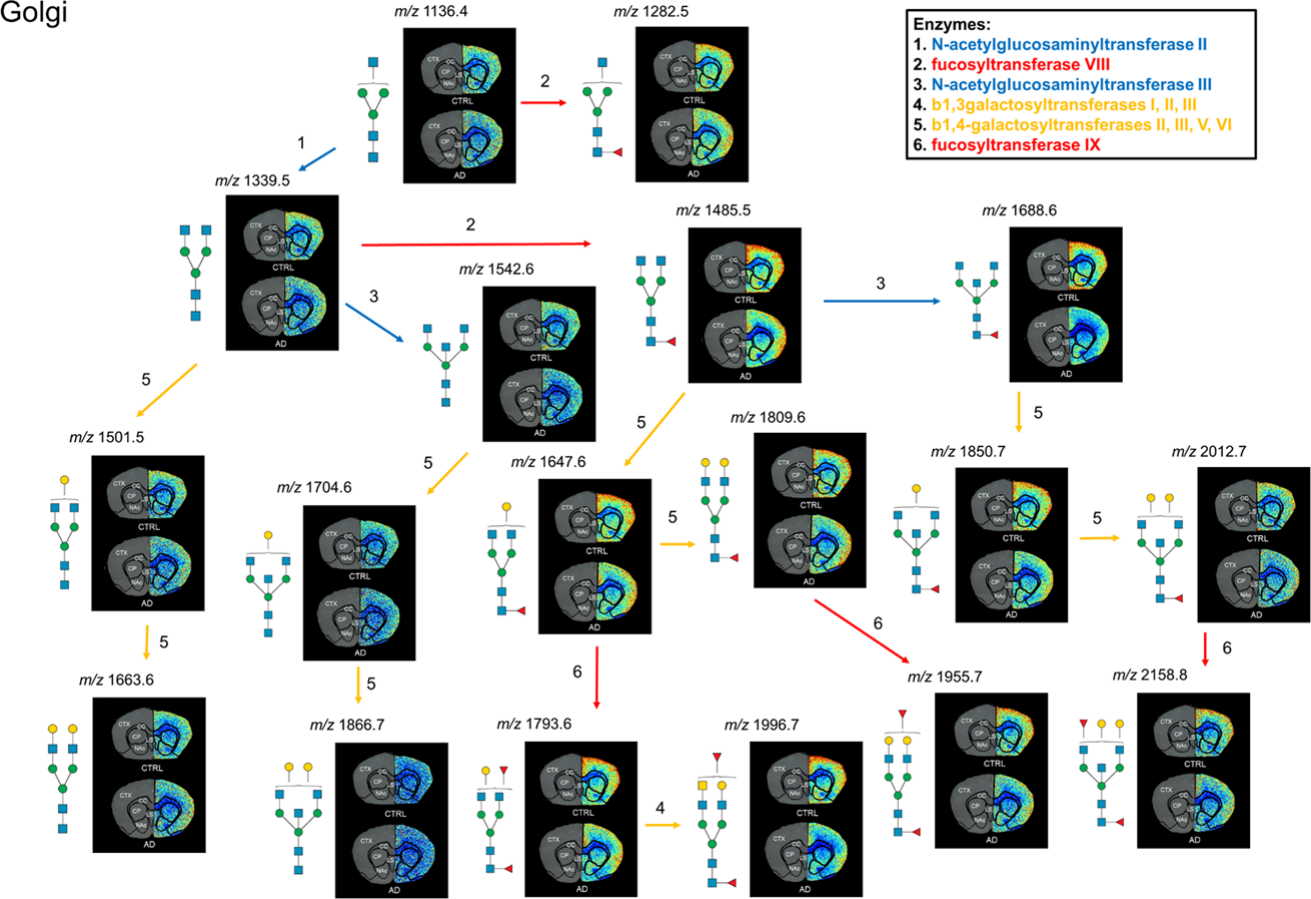
Biosynthetic pathway of *N*-glycans in the mouse CTX. Biantennary and GlcNAc-bisecting *N*-glycans with fucose showed the most significant alterations between the transgenic AD and CTRL groups within the CTX. Symbols for *N*-glycan monosaccharides: green circle—mannose, yellow circle—galactose, blue square—*N*-acetylglucosamine (GlcNAc), yellow square—*N*-acetylgalactosamine (GalNAc), and red triangle—fucose.

When comparing CTRL and transgenic AD brains, there was a noticeable decrease in biantennary fucosylated *N*-glycans, such as Hex4dHex2HexNAc4, Hex5dHex2HexNAc4 and Hex4dHex2HexNAc5, in the outer layer of the CTX in the AD model (Figures 4, 5 and S8). These changes were evident in the biosynthetic pathway of *N*-glycans in the mouse CTX (Figure 5).^46^ Interestingly, GlcNAc-bisecting fucosylated *N*-glycans were also markedly decreased in the CTX of the AD model, whereas almost no differences were observed in both biantennary and GlcNAc-bisecting *N*-glycans without fucose, suggesting potential alterations related to fucosylation (Figure 5).

A similar study revealed that glycan expression levels in AD and normal brains were largely comparable, except for a reduction in *N*-glycans specifically within the frontal cortex of AD patients.^47^ Another recent study supported these findings, reporting an overall decrease in galactosylation, fucosylation, bisection and antennary glycan numbers in both asymptomatic and symptomatic AD samples compared to normal brain samples.^48^ This reduction may be attributed to the dysregulation of glycosyltransferases, such as alpha-1,3-mannosyl-glycoprotein 2-beta-*N*-acetylglucosaminyltransferase (MGAT1) and alpha-(1,6)-FUT8, which modify these specific *N*-glycans in the cortex of AD brains. Previous research on brain glycosylation has shown that high levels of fucosylation are prevalent across most brain regions, playing a crucial structural role.^49^ Functionally, fucosylated glycoproteins are involved in molecular transduction, highlighting their significance in essential cellular functions, such as cell signaling.^49,50^ These findings suggest that the reduced fucosylation levels observed in AD could indicate disturbances in cellular metabolism, potentially contributing to the formation of amyloid plaques in the CTX.

The analysis of tryptic peptides revealed no statistical differences between the CTRL and transgenic AD groups. Compared to LC-MS/MS, MALDI-MSI provides lower proteome coverage due to inherent limitations. Specifically, MALDI-MSI typically only detects a small subset of proteins/peptides that are highly abundant or well ionized, while lower abundance proteins/peptides are often undetected.^51,52^ This suggests that some low abundance peptides, potentially more biologically relevant, may be outcompeted by higher abundance or relatively well ionized peptides, thereby compromising their reliable detection (Table S5).^51^ In line with these limitations, we observed that highly abundant proteins/peptides, such as succinyl-CoA:3-ketoacid-coenzyme A transferase (SCOT1), myelin basic protein (MBP), actin (ACTB) and hemoglobin subunit alpha (HBA), were consistently among the most enriched in both CTRL and transgenic AD brains (Figure S9). These limitations should be considered and further optimized in future studies. Given the challenge of achieving high product ion signals for on-tissue MS/MS identification of tryptic peptides due to low precursor ion signals generated with MALDI, identifications were conducted using LC-MS/MS. Overall, our findings underscore the complex interplay of lipids, *N*-glycans and tryptic peptides in the brains of the AD model, particularly in the CTX region. This emphasizes the importance of comprehensive multiomics approaches for elucidating disease mechanisms. These insights provide a foundation for further research on biomolecular interactions in neurodegenerative diseases.

## Conclusions

Systematic development and validation of a tissue wash protocol utilizing ice-cold methanol and chloroform significantly improved comprehensive analysis of lipids (in dual polarity mode), *N*-glycans and tryptic peptides in FF brain tissue sections using MALDI-MSI. The methodological advancement provided valuable insights into biomolecular alterations associated with pathology in the transgenic AD mouse model as a proof of concept. Future studies should focus on further refining the protocol and expanding its application to investigate other neurodegenerative disorders using both preclinical models and human post-mortem samples.

## Supporting Information Available

LC-MS/MS proteomics preparation, LC-MS/MS acquisition and analysis; spray parameters used for matrix and enzyme deposition in the MALDI-MSI workflow; TimsTOF flex instrument settings used for the analysis of lipids (in dual polarity mode), *N*-glycans, and tryptic peptides; comparison of tissue washing protocols for *N*-glycan MALDI-MSI analysis; selected *m*/*z* features of lipids and *N*-glycans from MALDI-MSI, highlighting the top 20 statistically significant differences between the AD mouse model and CTRLs; selected abundant *m*/*z* features of tryptic peptides detected by MALDI-MSI matched with LC-MS/MS data; *N*-glycan ion images showing localization in specific brain regions, demonstrating successful optimization of *N*-glycan MALDI-MSI analysis on FF brain tissue; representative overall spectra of lipids (in dual polarity mode), *N*-glycans, and tryptic peptides from two consecutive technical replicates of coronal rat cerebellum sections; H&E-stained images of consecutive technical replicate sagittal mouse brain sections and coronal rat cerebellum sections with annotated regions; volcano plots demonstrating fold changes and *p*-values of putative *m*/*z* features across lipid, *N*-glycan, and tryptic peptide data sets between the AD and CTRL groups for each brain region of interest; representative on-tissue MS/MS analysis of a lipid, an *N*-glycan, and a tryptic peptide using collision-induced dissociation (CID) fragmentation; box plots displaying statistical differences in the log-transformed intensity of *m*/*z* features, focusing on the top 20 most significant features; representative ion images of the top 20 most significant *m*/*z* features from lipids and *N*-glycans between the AD mouse model and CTRL group; H&E-stained images of CTRL (*n* = 5) and AD (*n* = 5) brain tissue sections with annotations for specific regions alongside representative ion images of SHexCer species and biantennary fucosylated *N*-glycans in the CTX, showing significant differences between the AD model and CTRL group; and comparison of normalized intensities of selected abundant tryptic peptides between CTRL and AD groups (PDF)
List of sequences (xlsx)
