# Supplementary material for: Comprehensive Approach for Sequential MALDI-MSI Analysis of Lipids, N-Glycans, and Peptides in Fresh-Frozen Rodent Brain Tissues
Source: Anal Chem. 2025 Jan 9;97(2):1338–46. doi: 10.1021/acs.analchem.4c05665 (PMC11755403; doi:10.1021/acs.analchem.4c05665)
Supplement: Supplementary file 1 — ac4c05665_si_001.pdf [file ac4c05665_si_001.pdf]

Supporting Information

Comprehensive Approach for Sequential MALDI-MSI Analysis of Lipids, *N*-Glycans and Peptides in Fresh-Frozen Rodent Brain Tissues

Yea-Rin Lee[1], Ibrahim Kaya[1], Elin Wik[2], Sooraj Baijnath[1,3], Henrik Lodén[1], Anna Nilsson[1], Xiaoqun Zhang[4], Dag Sehlin[2], Stina Syvänen[2], Per Svenningsson[4], Per E. Andrén[1*]

1. Department of Pharmaceutical Biosciences, Spatial Mass Spectrometry, Science for Life Laboratory, Uppsala University, Uppsala SE-75124, Sweden
2. Department of Public Health and Caring Sciences, Uppsala University, Uppsala SE-75237, Sweden
3. Integrated Molecular Physiology Research Initiative, School of Physiology, Faculty of Health Sciences, University of the Witwatersrand, Johannesburg 2017, South Africa
4. Department of Clinical Neuroscience, Karolinska Institute, Stockholm SE-17177, Sweden

*Corresponding Author

Per E. Andrén

Department of Pharmaceutical Biosciences, Spatial Mass Spectrometry, Science for Life Laboratory, Uppsala University, Uppsala SE-75124, Sweden
Email, per.andren@uu.se
Phone, +46-70 167 9334

Supplementary Methods

LC-MS/MS proteomics preparation. FF sagittal brain sections, encompassing most of the brain regions of interest, were additionally cryo-sectioned at a thickness of 12 µm and direCTRLy placed into 1.5 mL Eppendorf Protein LoBind tubes (three consecutive sections per tube, with two replicate tubes prepared). 100 µL of EasyPep lysis buffer (Thermo Fisher Scientific; A45735) was added to each tube and mixed by vigorously pipetting up and down. The tubes were briefly vortexed and sonicated for 15 min to achieve tissue homogenization. Next, the tubes were boiled at 98 °C for 30 min in a Thermomixer (Eppendorf Thermomixer Compact 5350, Hamburg, Germany) at 1300 rpm, followed by incubation at 80 °C for 1 h. After cooling to room temperature, the tubes were centrifuged (Centrifuge 5804 R, Eppendorf, Hamburg, Germany) at 14,000 rpm for 30 min at 4 °C and the supernatant was collected into new tubes. Subsequently, 50 µL of 50 mM ammonium bicarbonate (AB) was added to each tube and protein concentrations were measured using the Bradford assay [1].

Sample purification and protein digestion were performed using filter-aided sample preparation (FASP) as previously described with some modifications [2]. Briefly, approximately 20 µg of protein was placed on a centrifugal filter unit (Microcon-30 kDa; Merck, Darmstadt, Germany). The sample was washed with 50 µL of 8 M urea buffer on the filter and then centrifuged at 14,000 rpm for 15 min at 4 °C. Sample reduction was performed with 5 µL of 0.2 M dithiothreitol (DTT) at room temperature for 1 h, followed by alkylation with 5.6 µL of 0.275 M 2-chloroacetamide (CAA) at room temperature in the dark for 30 min. Before tryptic digestion (enzyme-protein ratio 1:50 (w/w)), the sample was washed with 50 µL of 50 mM AB and centrifuged at 14,000 rpm for 10 min. Tryptic digestion was performed overnight in a Thermomixer set to 500 rpm at 37 °C. The digested peptides were collected by washing the filter with 50 µL of 50 mM AB and centrifuging twice at 14,000 rpm for 10 min. Afterwards, 5.6 µL of 10% FA was added to achieve a final concentration of 1% (v/v) to stop digestion. Finally, tryptic peptides were desalted using C18 ZipTips (Millipore, Ireland) according to the manufacturer's instructions. The resulting samples were dried in a speedvac (Concentrator 5301, Eppendorf, Hamburg, Germany) at 45 °C and resuspended in 0.1% FA to a final concentration of 50 ng/mL of equivalent protein content.

LC-MS/MS acquisition and analysis. Desalted samples (1 µL injection) were analyzed using a timsTOF Q-TOF mass spectrometer equipped with a CaptiveSpray nano-electrospray ion source coupled to a nanoElute liquid chromatography (LC) system (Bruker Daltonics, Bremen, Germany). Peptides were loaded in mobile phase A (0.1% FA in water) and separated at a flow rate of 300 nL/min using a 42 min gradient on a PepSep™ C18 column (100 mm length, 0.075 mm inner diameter, 120 Å pore size, 1.9 µm particle size, Bruker Daltonics, Bremen, Germany). Mobile phase B consisted of 0.1% FA in ACN, with a gradient comprising 3 linear segments: from 5 to 27% in 36 min, from 27 to 40% in 2 min, and from 40 to 80% in 4 min. All separation processes were performed in an integrated toaster column oven set at 50 °C.

MS data were acquired in the data dependent acquisition-parallel accumulation and serial fragmentation (DDA-PASEF) mode, consisting of 4 PASEF MS/MS scans [3]. The capillary voltage was set to 1500 V, and MS and MS/MS spectra were acquired over a *m/z* range of 100 to 1700 with an ion mobility range (1/K0) from 0.6 to 1.6 V.s/cm^2. The ramp and accumulation time were set to 100 ms to achieve a duty cycle close to 100% and a total cycle time of 0.53 s. The collision energy was ramped linearly as a function of mobility from 59 eV at 1/K0 = 1.6 V.s/cm^2 to 20 eV at 1/K0 = 0.6 V.s/cm^2. Precursors with charge states

from 0 to 5 were selected with a target value of 20,000 and an intensity threshold of 2500. Each sample was run in duplicate.

DDA raw files were processed using PEAKS Studio (v.11, Bioinformatics Solution Inc., Waterloo, ON, Canada) with a *de novo* sequencing-assisted database search for sensitive and accurate peptide identification [4]. The precursor mass tolerance and fragment ion mass tolerance were set to 10 ppm and 0.05 Da, respectively, and two missed cleavages were allowed. Carbamidomethylation was set as a fixed modification, whereas oxidation was set as a variable modification, with a maximum of two variable modifications per peptide. Processed DDA data were extracted from PEAKS Studio. Singly charged *m/z* values from MALDI-MSI data were manually aligned with those calculated from doubly charged peptides identified by LC-MS/MS, with a peptide mass tolerance of +/- 7 ppm. Peptide sequences were searched against the reviewed (Swiss-Prot) database exclusively using the peptide search tool provided (https://www.uniprot.org/) [5].

Table of Contents

Table S1. Spray parameters used for matrix and enzyme deposition in the MALDI-MSI workflow.

Table S2. timsTOF flex instrument settings used for the analysis of lipids (in dual polarity mode), *N*-glycans and tryptic peptides.

Table S3. Comparison of tissue washing protocols for *N*-glycan MALDI-MSI analysis.

Table S4. Selected *m/z* features of lipids and *N*-glycans from MALDI-MSI, highlighting the top 20 statistically significant differences between the AD mouse model and CTRLs.

Table S5. Selected abundant *m/z* features of tryptic peptides detected by MALDI-MSI, matched with LC-MS/MS data.

Figure S1. *N*-glycan ion images showing localization in specific brain regions, demonstrating the successful optimization of *N*-glycan MALDI-MSI analysis on FF brain tissue.

Figure S2. Representative overall spectra of lipids (in dual polarity mode), *N*-glycans and tryptic peptides from two consecutive technical replicates of coronal rat cerebellum sections.

Figure S3. H&E-stained images of consecutive technical replicate sagittal mouse brain sections (CTRL) and coronal rat cerebellum sections (CTRL) with annotated regions.

Figure S4. Volcano plots demonstrating fold changes and *p*-values of putative *m/z* features across lipid, *N*-glycan and tryptic peptide datasets between the AD and CTRL groups for each brain region of interest.

Figure S5. Representative on-tissue MS/MS analysis of a lipid, an *N*-glycan, and a tryptic peptide using collision-induced dissociation (CID) fragmentation.

Figure S6. Box plots displaying statistical differences in the log-transformed intensity of *m/z* features, focusing on the top 20 most significant features.

Figure S7. Representative ion images of the top 20 most significant *m/z* features from lipids and *N*-glycans between the AD mouse model and CTRL group.

Figure S8. H&E-stained images of CTRL (*n*=5) and AD (*n*=5) brain tissue sections with annotations for specific regions alongside representative ion images of SHexCer species and biantennary fucosylated *N*-glycans in the CTX, showing significant differences between the AD model and CTRL group.

Figure S9. Comparison of normalized intensities of selected abundant tryptic peptides between the CTRL and AD groups.

Table S1. Spray parameters utilized for the deposition of matrices and enzymes in the MALDI-MSI workflow.

**Parameters**	**Lipids**	***N*-glycans**	**Peptides**	**Matrix**
Concentration	7.5 mg/mL norharmane	0.05 μg/μL PNGase F	0.1 μg/μL trypsin gold	7 mg/mL CHCA
Temperature (°C)	60	50	45	77
Flow rate (μL/min)	70	15	15	70
Pressure (psi)	9	9	9	9
Velocity (mm/min)	1200	1200	1200	1100
Track spacing (mm)	2	2.5	2.5	2.5
Number of passes	15	35	20	10
Pattern	CC	CC	CC	CC
Drying time (sec)	0	0	0	0

Abbreviations: CHCA, a-Cyano-4-hydroxycinnamic acid; PNGase F, Peptide-*N*-Glycosidase F.

Table S2. The timsTOF flex instrument settings used for the analysis of lipids (in dual polarity mode), *N*-glycans and tryptic peptides.

Parameters	Lipids	*N*-glycans	Peptides
**(1) MS settings**			
Scan begin	300.0 *m/z*	900.0 *m/z*	700.0 *m/z*
Scan end	2000.0 *m/z*	3200.0 *m/z*	3200.0 *m/z*
Ion polarity	Negative & Positive	Positive	Positive
Scan mode	MS	MS	MS
Shots	100	200	200
Frequency	10000 Hz	10000 Hz	10000 Hz
Power	60%	60%	60%
Laser	30, 30 µm (x, y) for negative<br>30, 30 µm (x, y spot offset) for positive	30, 30 µm (x, y)	30, 30 µm (x, y)
Raster	60, 60 µm	50, 50 µm	50, 50 µm
**(2) Tune settings**			
Transfer			
MALDI plate offset	50.0 V	50.0 V	50.0 V
Deflection 1 delta	70.0 V	70.0 V	70.0 V
Funnel 1 RF	350.0 Vpp	500.0 Vpp	500.0 Vpp
isCID energy	0.0 eV	0.0 eV	0.0 eV
Funnel 2 RF	350.0 Vpp	500.0 Vpp	500.0 Vpp
Multipole RF	350.0 Vpp	500.0 Vpp	500.0 Vpp
Collision cell			
Collision energy	10.0 eV	10.0 eV	10.0 eV

Collision RF	2500.0 Vpp	4000.0 Vpp	4000.0 Vpp
Quadrupole			
Ion energy	5.0 eV	5.0 eV	5.0 eV
Low mass	300.0 *m/z*	900.0 *m/z*	700.0 *m/z*
Focus pre TOF			
Transfer time	80.0 μs	180.0 μs	180.0 μs
Pre pulse storage	10.0 μs	25.0 μs	25.0 μs

Table S3. Comparison of tissue washing protocols optimized for *N*-glycan MALDI-MSI on FF brain tissue sections, focusing on minimizing delocalization and lipid signal interference while preserving *N*-glycan integrity. Representative ion images of commonly known *N*-glycans demonstrate the delocalization issue caused by inefficient tissue washing.

Protocol	Temperature	Washing details	De-localization	Lipid MALDI-MSI signal detected	*N*-glycan MALDI-MSI signal detected
Gradual ethanol washing (100%, 95%, 70%) + Carnoy's solution (60% ethanol/ 30% chloroform/ 10% acetic acid; v/v/v)	Room temperature	- Ethanol washing: 1 min x 3 times<br>- Carnoy's washing: 2 min	Yes	No	Yes
Gradual ethanol washing	Room temperature &<br>Ice-cold	- Ethanol washing: 1 min x 3 times	Yes	Yes	Yes
Gradual ethanol washing + Formalin fixation (10%)	Ice-cold	- Ethanol washing: 1 min x 3 times<br>- Formalin fixation: 5 min	Yes	Yes	Yes
Methanol fixation + Chloroform washing	Ice-cold	- Methanol fixation: 1 min x 2 times<br>- Chloroform washing: 30 sec x 2 times	No	No	Yes

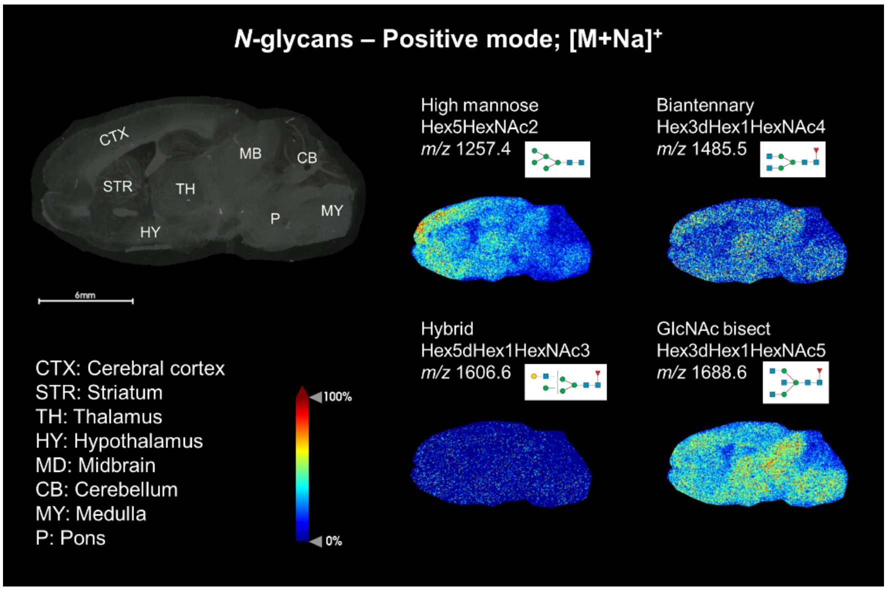

Table S4. List of the top 20 most significant *m/z* features for lipids and *N*-glycans with mass errors of < 2 ppm and < 6 ppm, respectively. These features, selected for their high significance between the AD mouse model and the CTRL group in the cerebral cortex, were used in further statistical analysis. Lipid and *N*-glycan identities were confirmed through mass accuracy comparison with published data and/or on-tissue MS/MS fragmentation using collision-induced dissociation (CID), performed only when the peak had sufficient intensity for isolation and fragmentation.

No	Species Assignment	Type (family)	Ion Type	Formula	*m/z* Theoretical	*m/z* Experimental	Error (ppm)	Identification
				**Lipids – Dual mode**				
1	PE-O(38:3)	Glycerophospholipids	$[M-H]^-$	C43H82NO7P	754.576	754.575	-0.42	On-tissue MS/MS
2	PE-O(40:8)	Glycerophospholipids	$[M-H]^-$	C45H76NO7P	772.529	772.528	-0.34	On-tissue MS/MS
3	PE-P(40:6)	Glycerophospholipids	$[M-H]^-$	C45H78NO7P	774.544	774.544	-0.66	On-tissue MS/MS & Mass match with published data [6]
4	PE-O(40:5)	Glycerophospholipids	$[M-H]^-$	C45H82NO7P	778.576	778.575	-1.18	On-tissue MS/MS
5	SHexCer(d36:1)	Sphingolipids	$[M-H]^-$	C42H81NO11S	806.546	806.546	0.05	On-tissue MS/MS & Mass match with published data [7]
6	PC(36:1)	Glycerophospholipids	$[M+K]^+$	C44H86NO8PK	826.572	826.572	0.17	On-tissue MS/MS & Mass match with published data [6]
7	PS(40:6)	Glycerophospholipids	$[M-H]^-$	C46H78NO10P	834.529	834.529	-0.07	On-tissue MS/MS & Mass match with published data [6]
8	SM(d42:2)	Sphingolipids	$[M+K]^+$	C47H93N2KO6P	851.640	851.639	-1.73	On-tissue MS/MS & Mass match with published data [6]
9	HexCer(t42:2)	Sphingolipids	$[M+K]^+$	C48H91NO9K	864.633	864.633	0.20	On-tissue MS/MS & Mass match with published data [6]
10	PC(40:6)	Glycerophospholipids	$[M+K]^+$	C48H84NO8PK	872.557	872.557	-0.02	On-tissue MS/MS & Mass match with published data [6]
11	SHexCer(t40:2)	Sphingolipids	$[M-H]^-$	C46H87NO12S	876.588	876.588	0.77	On-tissue MS/MS & Mass match with published data [6]
12	SHexCer(t40:1)	Sphingolipids	$[M-H]^-$	C46H89NO12S	878.603	878.602	-0.99	On-tissue MS/MS & Mass match with published data [7]
13	SHexCer(d42:3)	Sphingolipids	$[M-H]^-$	C48H89NO11S	886.608	886.608	-0.85	On-tissue MS/MS & Mass match with published data [6]
14	SHexCer(t42:3)	Sphingolipids	$[M-H]^-$	C48H89NO12S	902.603	902.603	-0.63	On-tissue MS/MS & Mass match with published data [6]
15	SHexCer(t42:2)	Sphingolipids	$[M-H]^-$	C48H91NO12S	904.619	904.618	-1.35	On-tissue MS/MS & Mass match with published data [7]
16	SHexCer(t43:2)	Sphingolipids	$[M-H]^-$	C49H93NO12S	918.635	918.634	-0.95	On-tissue MS/MS
17	SHexCer(t44:2)	Sphingolipids	$[M-H]^-$	C50H95NO12S	932.650	932.650	-0.67	On-tissue MS/MS
18	GM3(36:1)	Sphingolipids	$[M-H]^-$	C59H108N2O21	1179.737	1179.738	0.53	On-tissue MS/MS & Mass match with published data [6]
19	GM3(38:1)	Sphingolipids	$[M-H]^-$	C61H112N2O21	1207.769	1207.768	-0.48	On-tissue MS/MS & Mass match with published data [6]
20	GM2(36:1)	Sphingolipids	$[M-H]^-$	C67H121N3O26	1382.817	1382.816	-0.18	On-tissue MS/MS & Mass match with published data [6]
				***N*-glycans – Positive mode**				
1	Hex3HexNAc3	Biantennary	$[M+Na]^+$	C42H71N3O31	1136.396	1136.401	4.14	On-tissue MS/MS
2	Hex3HexNAc4	Biantennary	$[M+Na]^+$	C50H84N4O36	1339.476	1339.479	2.54	On-tissue MS/MS & Mass match with published data [8, 9]
3	Hex6HexNAc2	High mannose	$[M+Na]^+$	C52H88N2O41	1419.476	1419.480	3.24	On-tissue MS/MS & Mass match with published data [8, 9]
4	Hex4HexNAc4	Biantennary	$[M+Na]^+$	C56H94N4O41	1501.529	1501.531	1.86	On-tissue MS/MS & Mass match with published data [8, 9]
5	Hex3HexNAc5	GlcNAc bisect	$[M+Na]^+$	C58H97N5O41	1542.555	1542.559	2.72	On-tissue MS/MS & Mass match with published data [8, 9]
6	Hex7HexNAc2	High mannose	$[M+Na]^+$	C58H98N2O46	1581.528	1581.534	3.67	On-tissue MS/MS & Mass match with published data [8, 9]
7	Hex4dHex1HexNAc4	Biantennary	$[M+Na]^+$	C62H104N4O45	1647.587	1647.593	3.76	Mass match with published data [8, 9]

8	Hex3dHex1HexNAc5	GlcNAc bisect	$[M+Na]^+$	C64H107N5O45	1688.613	1688.618	2.66	Mass match with published data [8, 9]
9	Hex4HexNAc5	GlcNAc bisect	$[M+Na]^+$	C64H107N5O46	1704.608	1704.612	2.52	On-tissue MS/MS
10	Hex8HexNAc2	High mannose	$[M+Na]^+$	C64H108N2O51	1743.581	1743.588	3.96	On-tissue MS/MS & Mass match with published data [8, 9]
11	Hex4dHex2HexNAc4	Biantennary	$[M+Na]^+$	C68H114N4O49	1793.644	1793.650	2.95	Mass match with published data [8, 9]
12	Hex4dHex1HexNAc5	GlcNAc bisect	$[M+Na]^+$	C70H117N5O50	1850.666	1850.674	4.38	On-tissue MS/MS & Mass match with published data [8, 9]
13	Hex9HexNAc2	High mannose	$[M+Na]^+$	C70H118N2O56	1905.634	1905.644	5.09	Mass match with published data [8, 9]
14	Hex5dHex2HexNAc4	Biantennary	$[M+Na]^+$	C74H124N4O54	1955.697	1955.703	2.97	On-tissue MS/MS & Mass match with published data [8, 9]
15	Hex4dHex2HexNAc5	Biantennary	$[M+Na]^+$	C76H127N5O54	1996.724	1996.728	1.95	On-tissue MS/MS & Mass match with published data [8, 9]
16	Hex5dHex1HexNAc5	GlcNAc bisect	$[M+Na]^+$	C76H127N5O55	2012.719	2012.725	3.28	On-tissue MS/MS & Mass match with published data [8, 9]
17	Hex5dHex3HexNAc4	Biantennary	$[M+Na]^+$	C80H134N4O58	2101.755	2101.759	1.76	Mass match with published data [8, 9]
18	Hex5dHex2HexNAc5	GlcNAc bisect	$[M+Na]^+$	C82H137N5O59	2158.777	2158.783	2.78	On-tissue MS/MS & Mass match with published data [8, 9]
19	Hex5dHex3HexNAc5	GlcNAc bisect	$[M+Na]^+$	C88H147N5O63	2304.835	2304.835	0.39	Mass match with published data [8, 9]
20	Hex6dHex2HexNAc5	Multiantennary	$[M+Na]^+$	C88H147N5O64	2320.829	2320.834	2.11	Mass match with published data [8, 9]

Table S5. List of selected abundant *m/z* features of tryptic peptides with < 7 ppm mass error detected by MALDI-MSI and matched with LC-MS/MS datasets. There were no statistically significant differences between the AD mouse model and CTRL group. Additional LC-MS/MS data are available in a separate Excel file.

**UniProt ID**	**Sequence**	***m/z* MALDI [M+H]$^+$**	***m/z* LC-MS/MS [M+H]$^+$**	**Error (ppm)**
MBP_MOUSE	GSGKVPWLK	971.566	971.567	-1.03
ACTB_MOUSE	AGFAGDDAPR	976.451	976.448	3.07
SCOT1_MOUSE	DGSVALASKPR	1100.604	1100.605	-0.91
ACTB_MOUSE	AVFPSIVGRPR	1198.707	1198.705	1.67
SPTN1_MOUSE	GVLDMGNSLLER	1303.670	1303.667	2.30
H4_MOUSE	DNLQGLTKPALR	1325.757	1325.753	3.02
MBP_MOUSE	YLATASTMDHAR	1336.639	1336.631	5.99
MBP_MOUSE	TQDENPVVHFFK	1460.725	1460.716	6.16
HBA_MOUSE	LGGHGAEYGAEALER	1529.740	1529.734	3.92
TBA1A_MOUSE	AVFVDLEPTVLDEVR	1701.912	1701.905	4.11
ACTB_MOUSE	VAPEEHPVLLTEAPLNPK	1954.066	1954.064	1.02

Figure S1. Representative *N*-glycan ion images for $[M+Na]^+$ showing the localization of *N*-glycans in specific brain regions, illustrating the feasibility of *N*-glycan MALDI-MSI analysis of FF brain tissue sections.

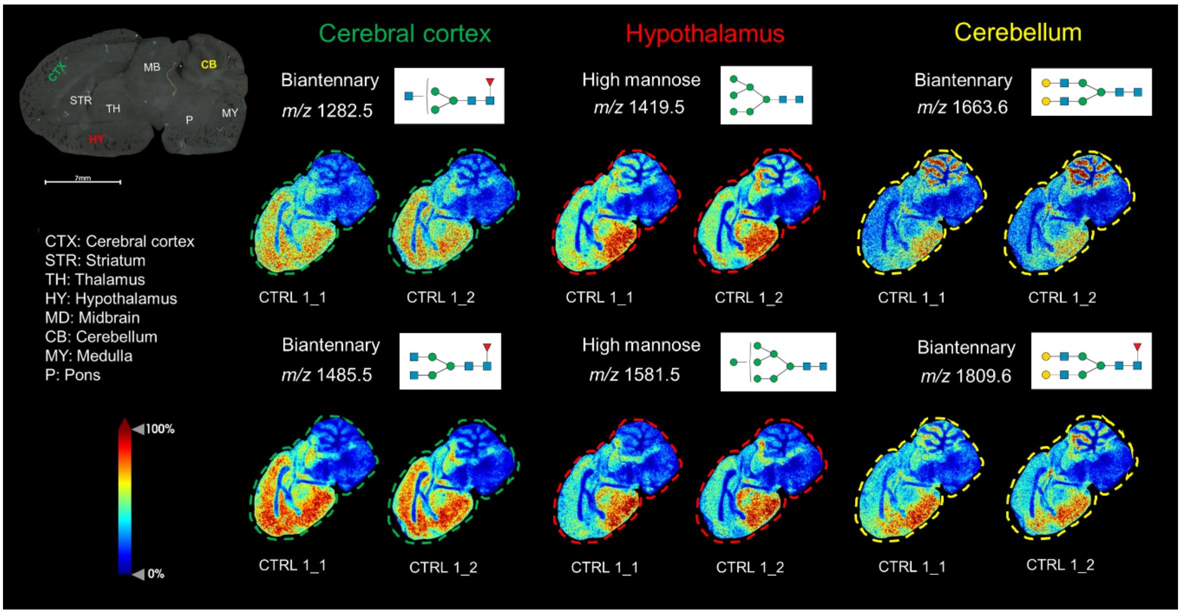

Figure S2. Representative overall spectra of lipids (in dual polarity mode), *N*-glycans and tryptic peptides from two consecutive technical replicates of coronal rat cerebellum sections (CTRL). The overall spectral patterns and number of *m/z* peaks were identical, confirming the applicability and reproducibility of the optimized method.

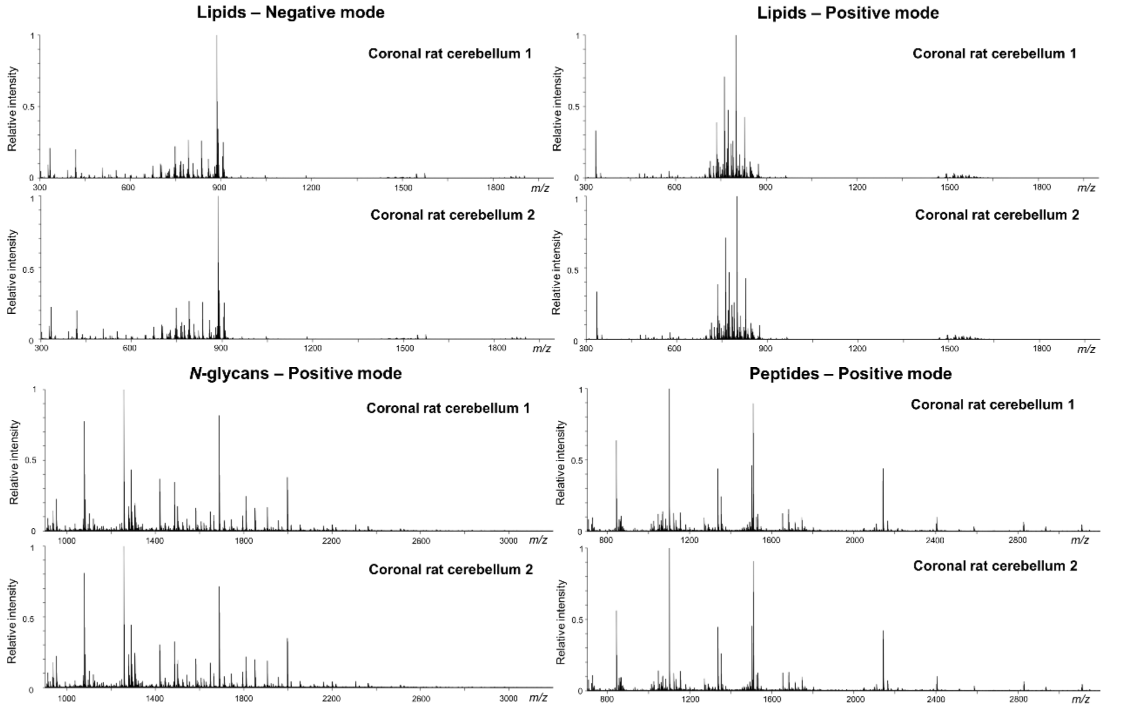

Figure S3. H&E-stained images of sagittal mouse brain sections (CTRL) and coronal rat cerebellum sections (CTRL) with annotated regions. Each brain sample, consisting of at least two consecutive technical replicate sections, was processed and measured in alternating order.

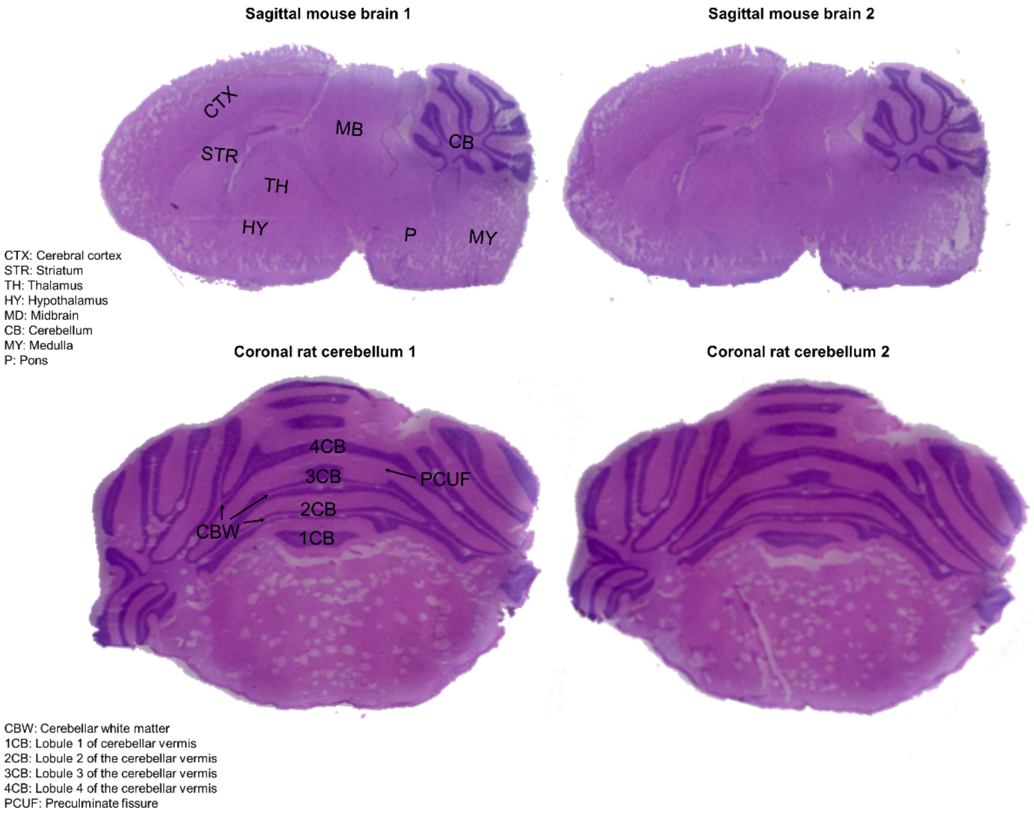

Figure S4. Volcano plots illustrating the abundance of putative *m/z* features (combining lipid, *N*-glycan and tryptic peptide datasets) and their fold changes ($\log_2$ scale) between the AD and CTRL groups, along with corresponding *p*-values ($-\log_{10}$ scale). The plots were generated for each region of interest as part of an untargeted analysis approach, highlighting significantly altered *m/z* features (> 1.3 fold change, $p < 0.05$) in red. Note that not all *m/z* features marked in red correspond to lipids, *N*-glycans or tryptic peptides. Abbreviations: CC: corpus callosum, CP: caudoputamen, NAc: nucleus accumbens, LS: lateral septal complex.

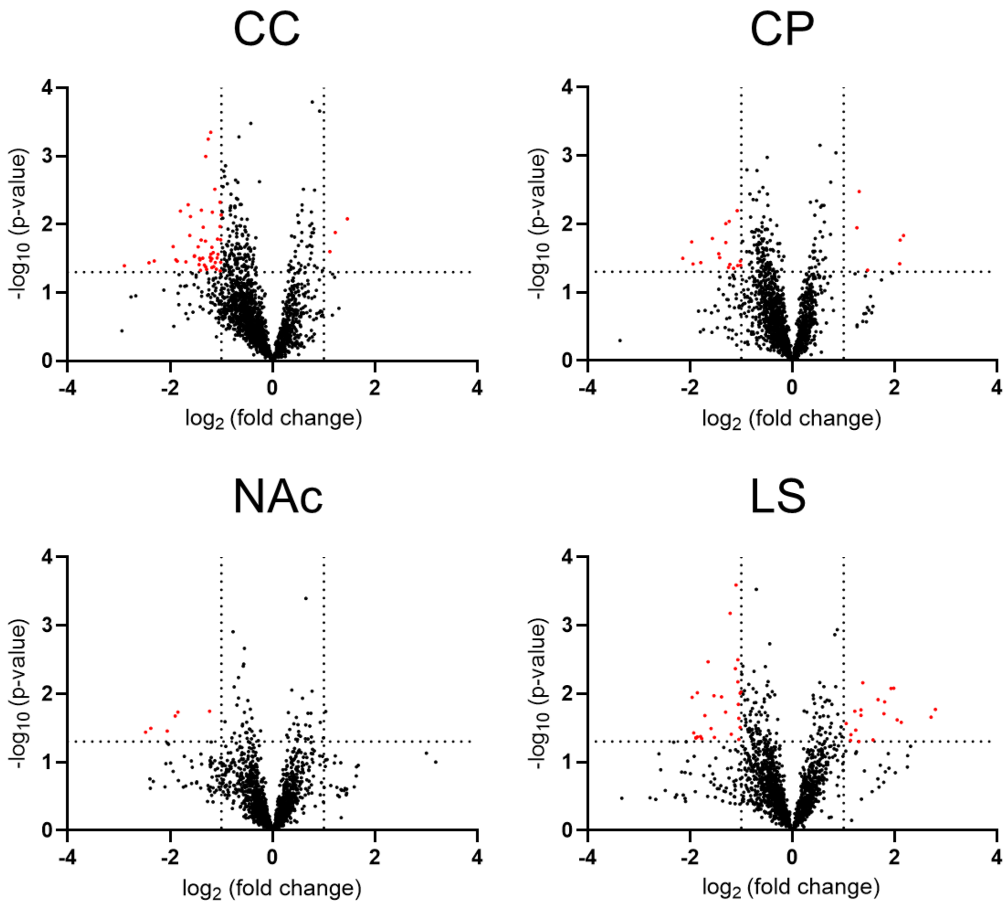

Figure S5. Representative on-tissue MS/MS analysis of a lipid, an *N*-glycan, and a tryptic peptide using collision-induced dissociation (CID) fragmentation. (A) Lipid fragments were identified by matching their experimental masses to theoretical messes from the precursor ion of PS (40:6) (*m/z* 834.5) using the LIPID MAPS database. (B) *N*-glycan fragments were annotated using GlycoWorkBench, based on the theoretical fragmentation masses of B, Y, C, and Z ions derived from the sodiated structure of Hex4HexNAc4 with a free end (*m/z* 1501.5). (C) The tryptic peptide with *m/z* 1198.7 was identified as 'AVFPSIVGRPR' using Mascot, which matched the theoretical b- and y- ion fragmentation masses from the SwissProt *Mus musculus* database.

A

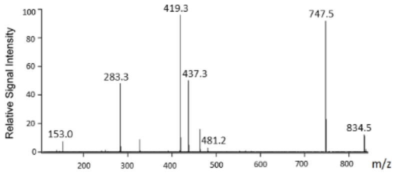

m/z	Fragment
153.0	glycerol-3-phosphate ion with loss of H2O
283.3	sn1 RCOO- ion
419.3	neutral loss of sn2 RCOOH group and serine
437.3	loss of sn2 acyl chain as ketene, RCH=C=O, and serine
481.2	loss of sn1 acyl chain as ketene, RCH=C=O, and serine
747.5	loss of serine from precursor ion

B

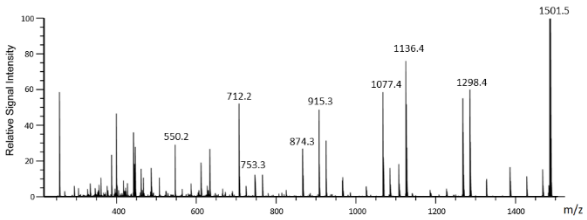

m/z	Fragment	Ions
550.2	BYY BY B BYY CZY CZY	Na
712.2	BYY BY BY BYY CZY CZ CZ	Na
753.3	BY BYY CZ CZY CYZ	Na
874.3	BY CZ	Na
915.3	BY BY BYY BY CZ CZ CZY YZ	Na
1077.4	BY B CZ	Na
1136.4	CY Y Y YY	Na
1298.4	C Y	Na

C

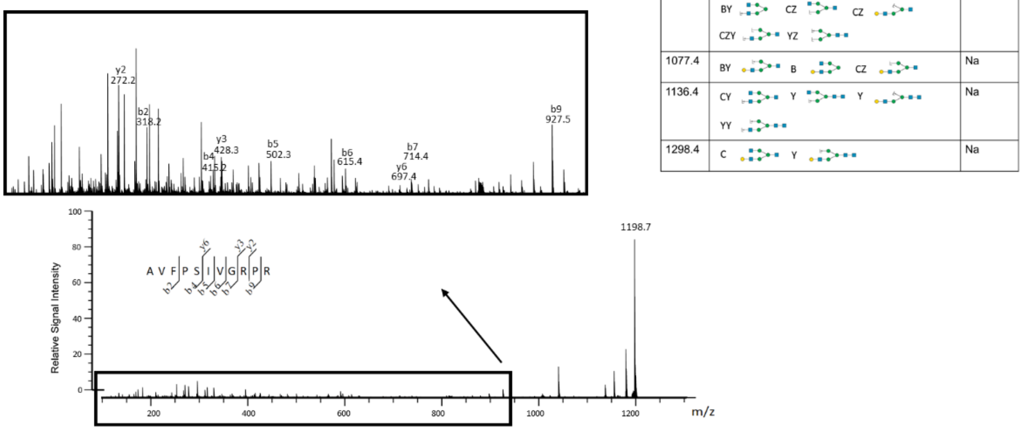

Figure S6. Box plots showing statistical differences in the log-transformed intensity of *m/z* features, focusing on the top 20 most significant features in the cerebral cortex of the AD model compared to the CTRL group. Statistical significance: * $p < 0.05$, ** $p < 0.01$, *** $p < 0.001$, **** $p < 0.0001$.

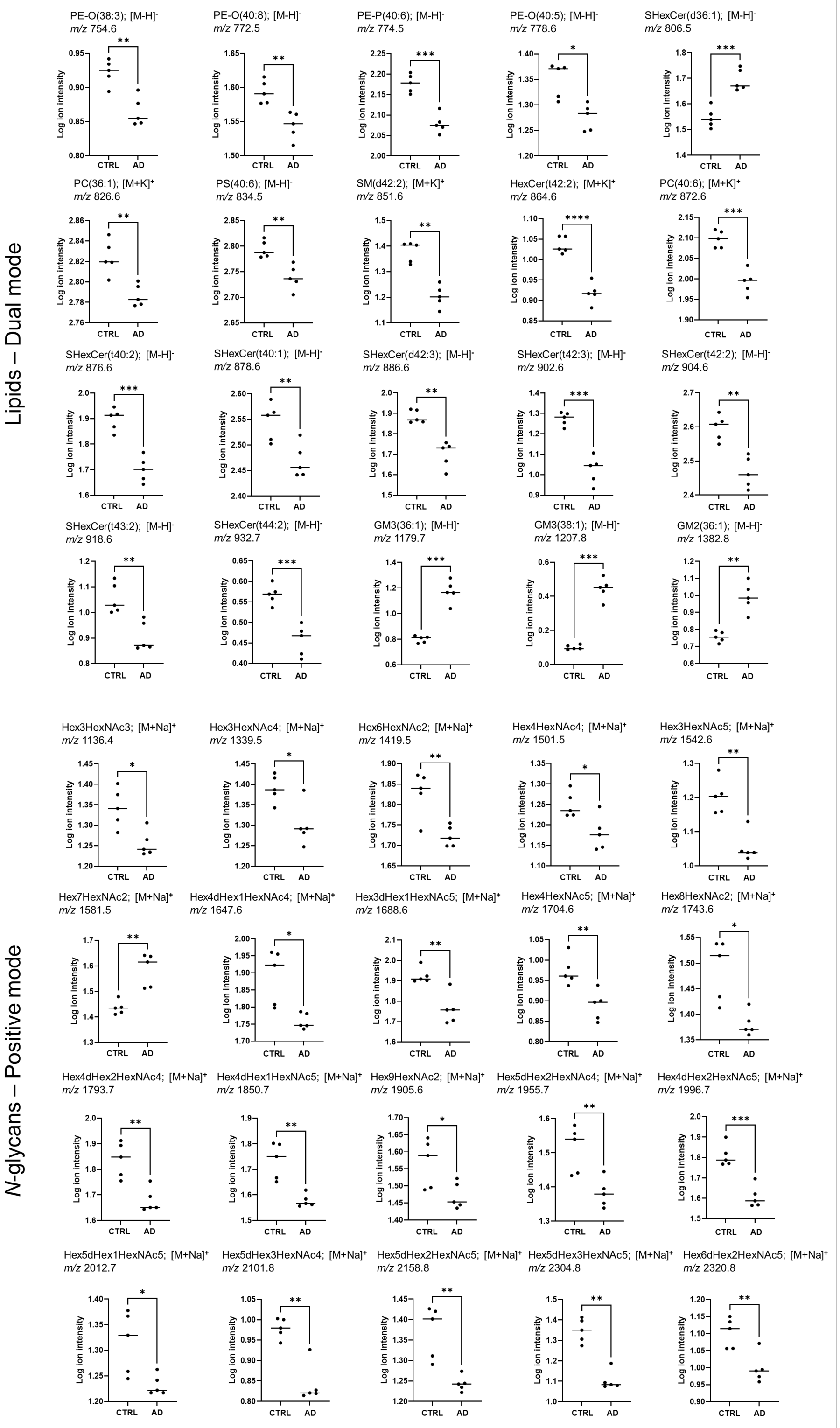

Figure S7. Representative ion images of the top 20 most significant *m/z* features from lipids and *N*-glycans, comparing the AD mouse model with the CTRL group. The images are listed in order from lowest to highest *m/z* values.

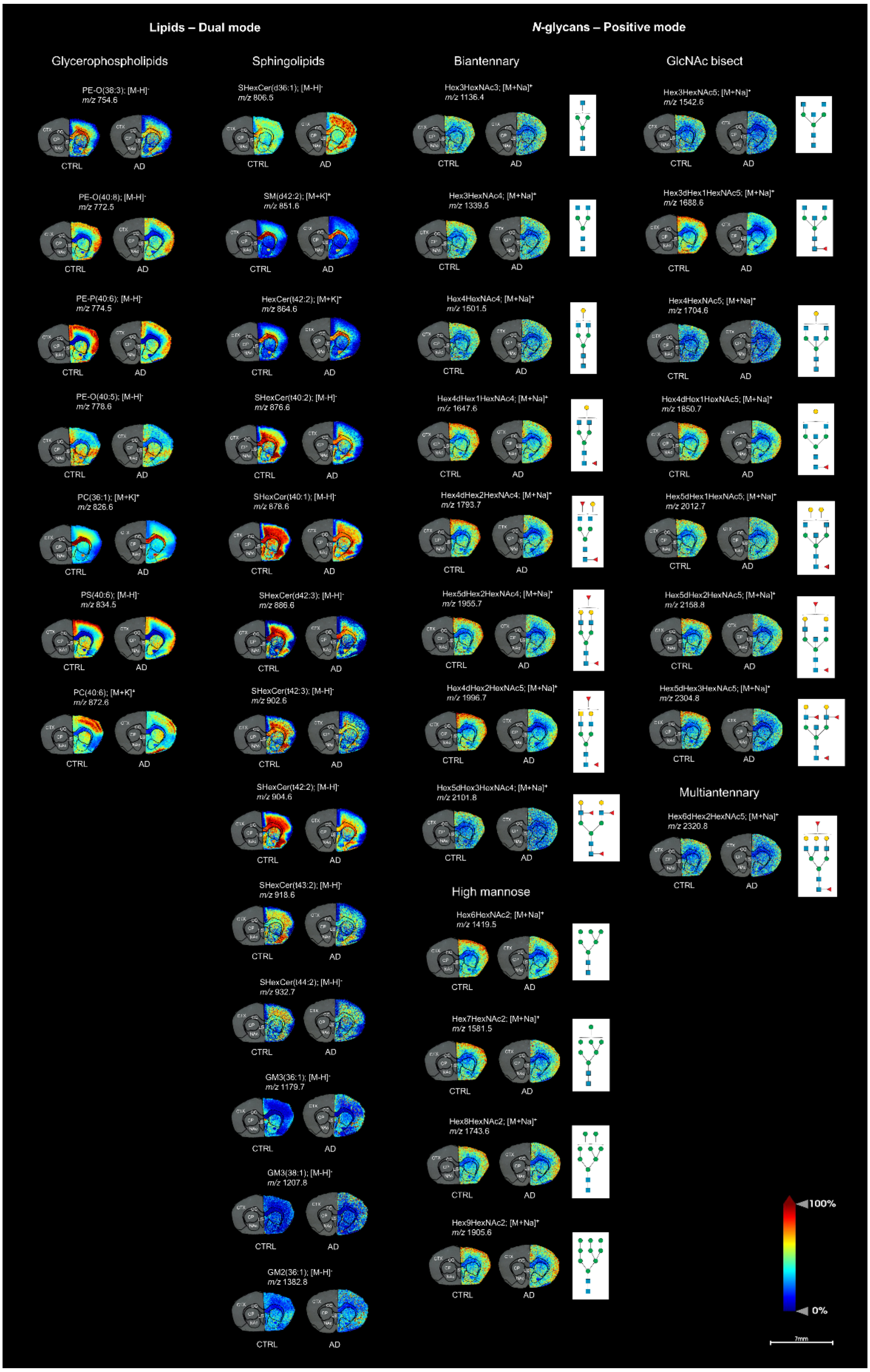

Figure S8. (A) H&E-stained images of CTRL (*n*=5) and AD (*n*=5) brain tissue sections with annotations for specific regions of interest: cerebral cortex (CTX), corpus callosum (CC), caudoputamen (CP), nucleus accumbens (ACB), lateral septal complex (LSX). (B) Representative ion images of SHexCer species and biantennary fucosylated *N*-glycans in the CTX illustrating significant differences between the AD mouse model and CTRL group.

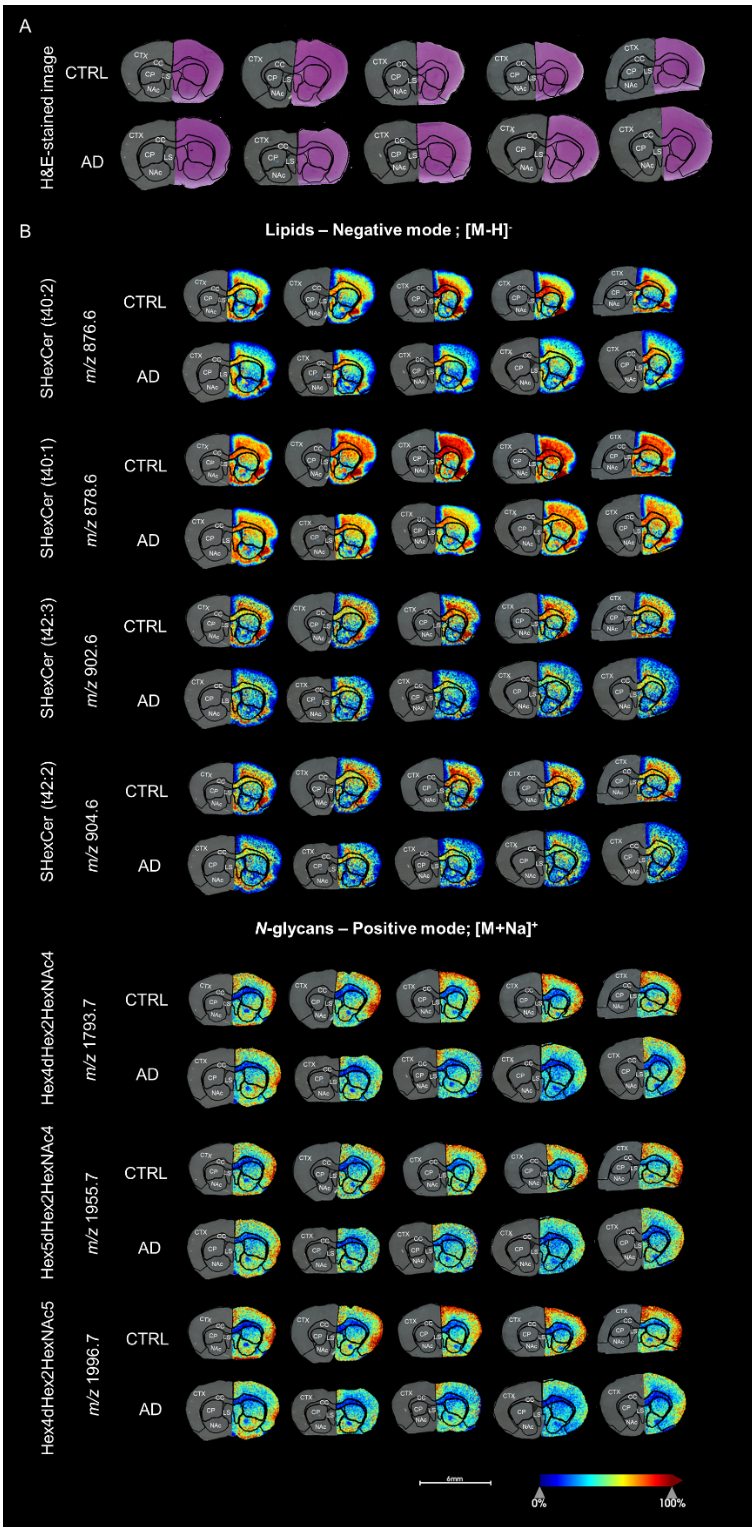

Figure S9. Overall normalized intensity of selected abundant *m/z* features of tryptic peptides detected by MALD-MSI in the CTRL and AD groups. The trend shows a decrease in their intensity in the AD mouse model (*n*=5) compared to the CTRL group (*n*=5). However, no statistically significant differences were observed.

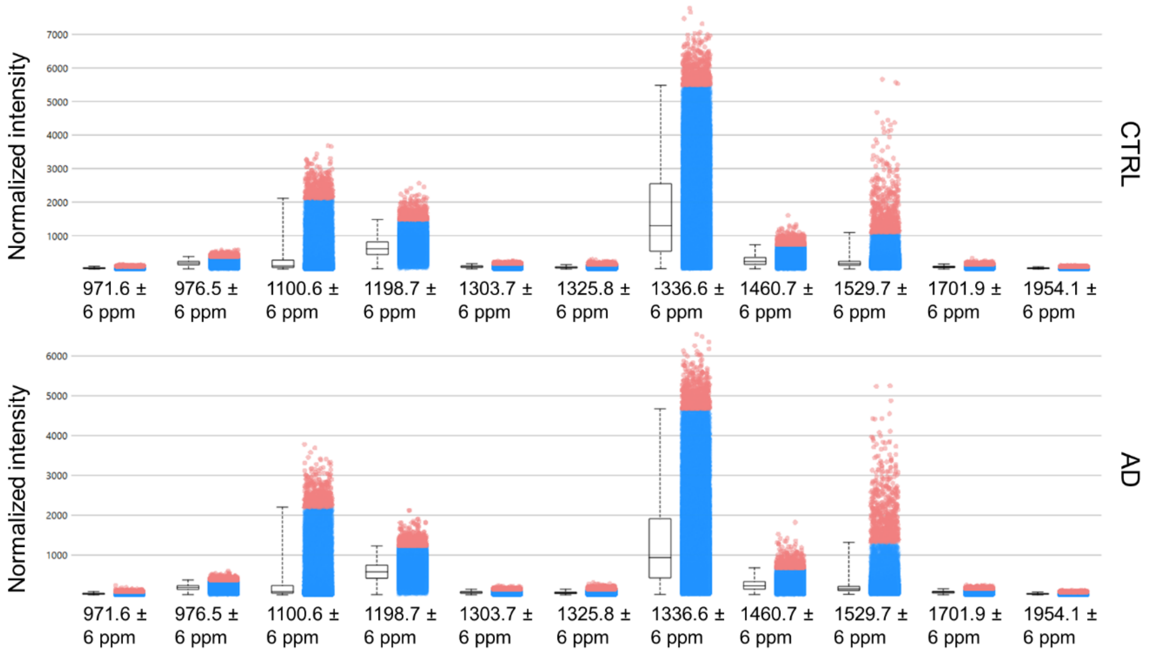

References

(1) Nouroozi, R. Determination of Protein Concentration Using Bradford Microplate Protein Quantification Assay. *Int Electron J Med* **2015**, *4*.

(2) Sandbaumhüter, F. A.; Nezhyva, M.; Andrén, P. E.; Jansson, E. T. Label-Free Quantitative Thermal Proteome Profiling Reveals Target Transcription Factors with Activities Modulated by MC3R Signaling. *Anal Chem* **2023**, *95* (41), 15400-15408.

(3) Meier, F.; Beck, S.; Grassl, N.; Lubeck, M.; Park, M. A.; Raether, O.; Mann, M. Parallel Accumulation-Serial Fragmentation (PASEF): Multiplying Sequencing Speed and Sensitivity by Synchronized Scans in a Trapped Ion Mobility Device. *J Proteome Res* **2015**, *14* (12), 5378-5387.

(4) Zhang, J.; Xin, L.; Shan, B.; Chen, W.; Xie, M.; Yuen, D.; Zhang, W.; Zhang, Z.; Lajoie, G. A.; Ma, B. PEAKS DB: de novo sequencing assisted database search for sensitive and accurate peptide identification. *Mol Cell Proteomics* **2012**, *11* (4), M111.010587.

(5) Chen, C.; Li, Z.; Huang, H.; Suzek, B. E.; Wu, C. H. A fast Peptide Match service for UniProt Knowledgebase. *Bioinformatics* **2013**, *29* (21), 2808-2809.

(6) Kaya, I.; Nilsson, A.; Luptáková, D.; He, Y.; Vallianatou, T.; Bjärterot, P.; Svenningsson, P.; Bezard, E.; Andrén, P. E. Spatial lipidomics reveals brain region-specific changes of sulfatides in an experimental MPTP Parkinson's disease primate model. *NPJ Parkinsons Dis* **2023**, *9* (1), 118.

(7) Zhang, Q.; Li, Y.; Sui, P.; Sun, X.-H.; Gao, Y.; Wang, C.-Y. MALDI mass spectrometry imaging discloses the decline of sulfoglycosphingolipid and glycerophosphoinositol species in the brain regions related to cognition in a mouse model of Alzheimer's disease. *Talanta* **2024**, *266*, 125022.

(8) Fülöp, A.; Marsching, C.; Barka, F.; Ucal, Y.; Pfänder, P.; Opitz, C. A.; Barka, G.; Hopf, C. Device-Controlled Microcondensation for Spatially Confined On-Tissue Digests in MALDI Imaging of N-Glycans. *Pharmaceuticals (Basel)* **2022**, *15* (11), 1356.

(9) Toghi Eshghi, S.; Yang, S.; Wang, X.; Shah, P.; Li, X.; Zhang, H. Imaging of N-Linked Glycans from Formalin-Fixed Paraffin-Embedded Tissue Sections Using MALDI Mass Spectrometry. *ACS Chem Biol* **2014**, *9* (9), 2149-2156.
